# Cross-modal correspondence enhances elevation localization in visual-to-auditory sensory substitution

**DOI:** 10.3389/fpsyg.2023.1079998

**Published:** 2023-01-26

**Authors:** Camille Bordeau, Florian Scalvini, Cyrille Migniot, Julien Dubois, Maxime Ambard

**Affiliations:** ^1^LEAD-CNRS UMR5022, Université de Bourgogne, Dijon, France; ^2^ImViA EA 7535, Université de Bourgogne, Dijon, France

**Keywords:** Virtual Acoustic Space, spatial hearing, sound spatialization, image-to-sound conversion, cross-modal correspondence, assistive technology, visual impairment, sound source localization

## Abstract

**Introduction:**

Visual-to-auditory sensory substitution devices are assistive devices for the blind that convert visual images into auditory images (or soundscapes) by mapping visual features with acoustic cues. To convey spatial information with sounds, several sensory substitution devices use a Virtual Acoustic Space (VAS) using Head Related Transfer Functions (HRTFs) to synthesize natural acoustic cues used for sound localization. However, the perception of the elevation is known to be inaccurate with generic spatialization since it is based on notches in the audio spectrum that are specific to each individual. Another method used to convey elevation information is based on the audiovisual cross-modal correspondence between pitch and visual elevation. The main drawback of this second method is caused by the limitation of the ability to perceive elevation through HRTFs due to the spectral narrowband of the sounds.

**Method:**

In this study we compared the early ability to localize objects with a visual-to-auditory sensory substitution device where elevation is either conveyed using a spatialization-based only method (Noise encoding) or using pitch-based methods with different spectral complexities (Monotonic and Harmonic encodings). Thirty eight blindfolded participants had to localize a virtual target using soundscapes before and after having been familiarized with the visual-to-auditory encodings.

**Results:**

Participants were more accurate to localize elevation with pitch-based encodings than with the spatialization-based only method. Only slight differences in azimuth localization performance were found between the encodings.

**Discussion:**

This study suggests the intuitiveness of a pitch-based encoding with a facilitation effect of the cross-modal correspondence when a non-individualized sound spatialization is used.

## 1. Introduction

Visual-to-auditory Sensory substitution devices (SSDs) are assistive tools for blind people. They convert visual information into auditory information in order to convey spatial information about the surrounding environment when vision is impaired. The visual-to-auditory conversion relies on the mapping of selected visual features with specific auditory cues. Visual information is usually acquired using a camera capturing the visual scene in front of the person. Then the scene converted into auditory information is transmitted to the user through soundscapes (or auditory images) delivered with headphones.

Various visual-to-auditory encodings are used by the existing visual-to-auditory SSDs to convey spatial information. Some of them use encoding schemes based on a Virtual Acoustic Space (VAS). A VAS consists in the simulation of a binaural acoustic signature of a virtual sound source located in a 3D space. In the context of visual-to-auditory SSDs, this is mainly used to simulate sound sources at the location of the obstacles. This simulation is achieved by spatializing the sound through the incorporation of spatial auditory cues in the original monophonic sound. Then a synthesized stereophonic signal simulating the distortions occurring while receiving the audio signal by the two ears is obtained. Among the SSDs used in localization experiments, the Synaestheatre (Hamilton-Fletcher et al., 2016a; Richardson et al., 2019), the Vibe (Hanneton et al., 2010) and the one presented by Mhaish et al. (2016) spatialize azimuth (lateral position) and elevation (vertical position). Other SSDs only spatialize the azimuth: the See differently device (Rouat et al., 2014), the one studied in Ambard et al. (2015), and the recent one presented in Scalvini et al. (2022).

The generation of a VAS is based on the reproduction of binaural acoustic cues related to the relative sound source location such as timing, intensity and spectral features (for an in-depth explanation of the auditory localization mechanisms see Blauert, 1996). Those features arise from audio signal distortions mainly caused by the reflection and absorption of the head, pinna and torso and are partly reproducible using Head-Related Transfer Functions (HRTFs). HRTFs are transfer functions characterizing these signal distortions as a function of the position of the sound source relatively to the two ears. They are usually obtained by conducting multiple binaural recordings with a sound source carefully placed in various positions while repeatedly producing the same sound.

Due to the technical difficulty in acquiring these recordings in good conditions, non-individualized HRTFs acquired in controlled conditions with another listener or a manikin are frequently used. However, these HRTFs failed to simulate the variability of individual-specific spectrum distortions that are related to individual morphologies (head, torso and pinna). Consequently, the localization of simulated sound sources using non-individualized HRTFs is often inaccurate with front-back and up-down confusions that are less resolvable (Wenzel et al., 1993), and a less perceptible externalization (Best et al., 2020). Nonetheless, due to the robustness of the binaural cues, azimuth localization accuracy is well preserved compared to the perception of elevation since azimuth perception relies less on the individual-specific spectrum distortions (Makous and Middlebrooks, 1990; Wenzel et al., 1993; Middlebrooks, 1999). Therefore, visual-to-auditory encodings only based on the creation of a VAS have the advantage to rely on acoustic cues that mimic natural acoustic features for sound source localization, nevertheless in practice the elevation perception can be impaired.

To compensate for this difficulty some visual-to-auditory SSDs use additional acoustic cues to convey spatial information. For instance, pitch modulation is often used to convey elevation location (Meijer, 1992; Abboud et al., 2014; Ambard et al., 2015). This mapping between elevation location and auditory pitch is based on the audiovisual cross-modal correspondence between pitch and elevation (see Spence, 2011 for a review on audiovisual cross-modal correspondences). Humans show a tendency to associate high pitch with high spatial locations and low pitch with low spatial locations. For example, they tend to exhibit faster response times in an audio-visual Go/No-Go task when the visual and auditory stimuli are congruent, i.e., higher pitch with higher visual location, and lower pitch with lower visual location (Miller, 1991). They also tend to discriminate more accurately and quickly the location of a visual stimulus (high *vs*. low location) when the pitch of a presented sound is congruent with the visual elevation (Evans and Treisman, 2011). Also, humans tend to respond to high pitch sounds with a high-located response button instead of a lower-located response button (Rusconi et al., 2006). The pitch-based encoding used in the vOICe SSD (Meijer, 1992) has been suggested somewhat intuitive in a recognition task (Stiles and Shimojo, 2015). Nevertheless, the main drawback of a pitch-based encoding is caused by the limitation of the abilities to perceive elevation through HRTFs due to the audio spectral narrowband (Algazi et al., 2001b). Although some acoustic cues for elevation perception are present in low frequencies below 3,500 Hz (Gardner, 1973; Asano et al., 1990), localization abilities are higher when the spectral content contains high frequencies above 4,000 Hz (Hebrank and Wright, 1974; Middlebrooks and Green, 1990). Since the ability to perceive the elevation through HRTFs is higher with broadband sounds containing high frequencies, the spectral content of the sound used in the visual-to-auditory encoding might modulate the perception of elevation through HRTFs. No study has directly compared encodings only based on HRTFs with encodings adding a pitch modulation and it remains unclear if the simulation of natural acoustic cues is less efficient for object localization than a more artificial sonification method using the cross-modal correspondence between pitch and elevation.

Many studies investigating static object localization abilities have already been conducted with blindfolded sighted persons using visual-to-auditory SSDs. Various types of tasks have already been used, for example discrimination tasks with forced choice (Proulx et al., 2008; Levy-Tzedek et al., 2012; Ambard et al., 2015; Mhaish et al., 2016; Richardson et al., 2019), grasping tasks (Proulx et al., 2008), index or tool pointing tasks (Auvray et al., 2007; Hanneton et al., 2010; Brown et al., 2011; Pourghaemi et al., 2018; Commère et al., 2020), or head-pointing tasks (Scalvini et al., 2022). Those studies showed the high potential of SSDs to localize an object and interact with it. However, long trainings were often conducted before the localization tasks to learn the visual-to-auditory encoding schemes: from 5 min in Pourghaemi et al. (2018) to 3 h in Auvray et al. (2007). On the contrary, in the study of Scalvini et al. (2022) the experimenter only explained verbally the encoding schemes to the participants.

Virtual environments are more and more used to investigate the abilities to perceive the environment with a visual-to-auditory SSD (Maidenbaum et al., 2014; Kristjánsson et al., 2016) since they allow a complete control of the experimental environment (e.g., number of objects, object locations...) (Maidenbaum and Amedi, 2019) and a more accurate assessment of localization abilities with precise pointing methods. They have been used in standardization tests to compare the abilities to interpret information provided by SSDs in navigation or localization tasks (Caraiman et al., 2017; Richardson et al., 2019; Jicol et al., 2020; Real and Araujo, 2021).

The current study aimed at investigating the intuitiveness of different types of visual-to-auditory encodings for the elevation in the context of object localization with a SSD. Therefore, we conducted a localization task in a virtual environment with blindfolded participants testing a spatialization-based encoding and a pitch-based encoding. This study also aimed at assessing whether a higher spectral complexity of the sound used in a pitch-based encoding could improve the localization performance. Therefore, 2 types of pitch-based encodings were investigated: one monotonic and one harmonic with 3 octaves. We measured the localization performance for the azimuth and for the elevation. For each of these measures, we studied the effect of the visual-to-auditory encoding before and after an audio-motor familiarization of short duration.

Since the audio spatialization method was not based on individualized HRTFs, and since the pitch-based encodings were not explained to the participants, localization performance for the elevation was expected to be impaired. However, a facilitation effect of the pitch-based encodings for the elevation localization accuracy was hypothesized. Among the two pitch-based encodings, a higher elevation localization accuracy was predicted with the harmonic encoding since the sound has a higher spectral complexity. Also, the intuitiveness of the azimuth perception for all the encodings was hypothesized since it is based on less individual-specific acoustic spatial cues than elevation perception.

## 2. Method

### 2.1. Participants

Thirty eight participants were divided into two groups: the Monotonic group (19, age: *M* = 25.5, *SD* = 3.04, 6 female, 19 right-handed) and the Harmonic group (19, age: *M* = 24.4, *SD* = 3.27, 10 female, 18 right-handed). No participant reported impairments of hearing or any history of psychiatric illness or neurological disorder. The experimental protocol was approved by the local ethical committee Comité d'Ethique pour la Recherche de Université Bourgogne Franche-Comté (CERUBFC-2021-12-21-050) and followed the ethical guidelines of the Declaration of Helsinki. Written informed consent was obtained from all the participants before the experiment. No monetary compensation was given to the participants.

### 2.2. Visual-to-auditory conversion in the virtual environment

The visual-to-auditory SSD used took place in a virtual environment created in UNITY3D and including the target to localize, a virtual camera, and a tracked pointing tool. Four HTC VIVE base stations were used to track the participants' head and the pointing tool on which HTC VIVE Trackers 2.0 were attached. Participants did not carry a headset and therefore could not explore visually the virtual environment. The pointing task can be separated in several steps that are explained in detail below: the virtual target placement, the video acquisition from a virtual camera, the video processing, the visual-to-auditory conversion and the participants' response collection using the pointing tool.

#### 2.2.1. Virtual target

**Figure 1:**
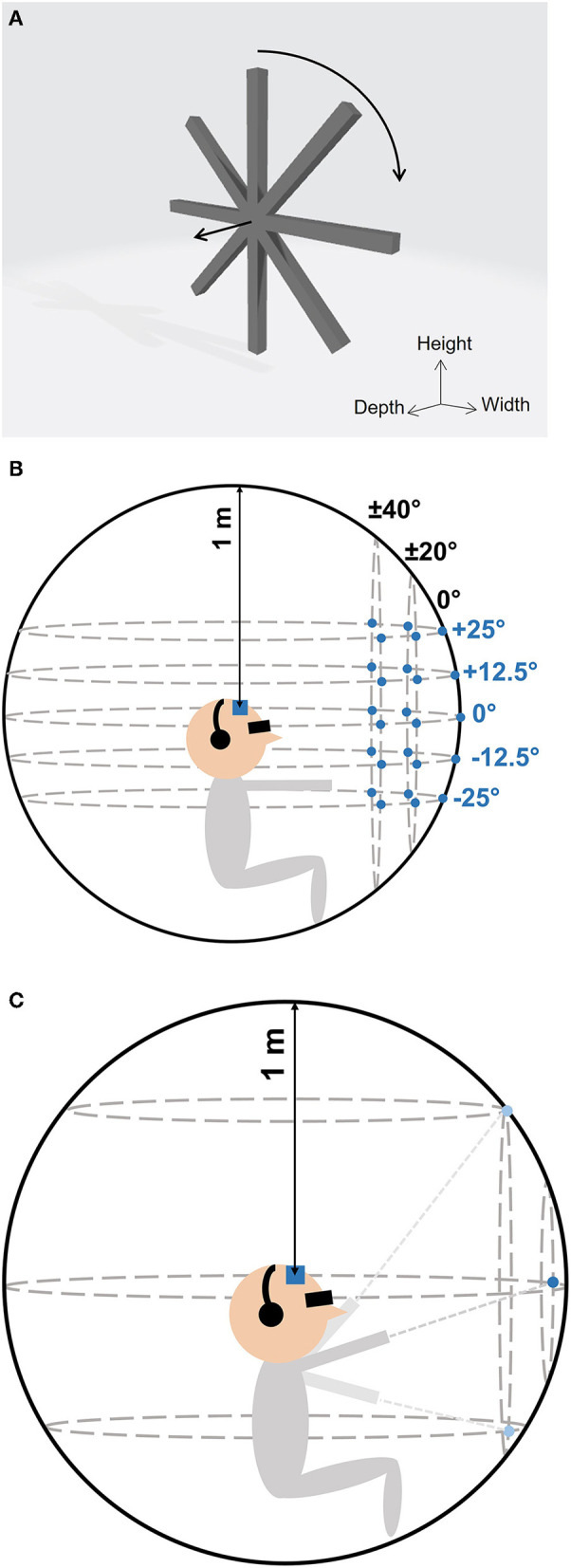
**(A)** The virtual target was a self-rotating 3D propeller shape. The straight arrow shows the forward axis facing the virtual camera. The circular arrow shows the self-rotation direction. **(B)** In each localization test, 25 target positions (blue circles) were tested, including 5 elevation positions (horizontal dotted ellipses) tested at 5 azimuth positions (vertical dotted ellipses). **(C)** In the familiarization session, participants placed the virtual target during 60 seconds on the grid by moving the pointing tool. Participants' head were tracked during the localization tests and familiarization sessions (blue square).

The virtual target that participants had to localize was a 3D propeller shape of 25 cm in diameter composed of 4 bars with a length of 25 cm and a rectangular section of 5 × 5 cm that was self-rotating at a speed of 10° per video frame (see Figure 1A). The use of an angular shaped target that is self-rotating generated a modification of successive video frames without changing the center position of the target. The orientation of the target was managed in order to continuously face the virtual camera while being displayed. Since participants could not see the virtual target, it was only perceivable through the soundscapes.

#### 2.2.2. Video acquisition

The virtual camera position was set at the beginning of each trial using the position of the head tracker attached on the participants' forehead. Images were acquired with a virtual camera with a field of view of 90 × 74° (Horizontal × Vertical) and a frame rate of 60 Hz. The resulting image was using a grayscale encoding (0–255 gray levels) of a depth map (0.2 m = 0, 5.0 m = 255) of the virtual scene although in this experiment we did not manipulate the depth parameter.

#### 2.2.3. Video processing

**Figure 2:**
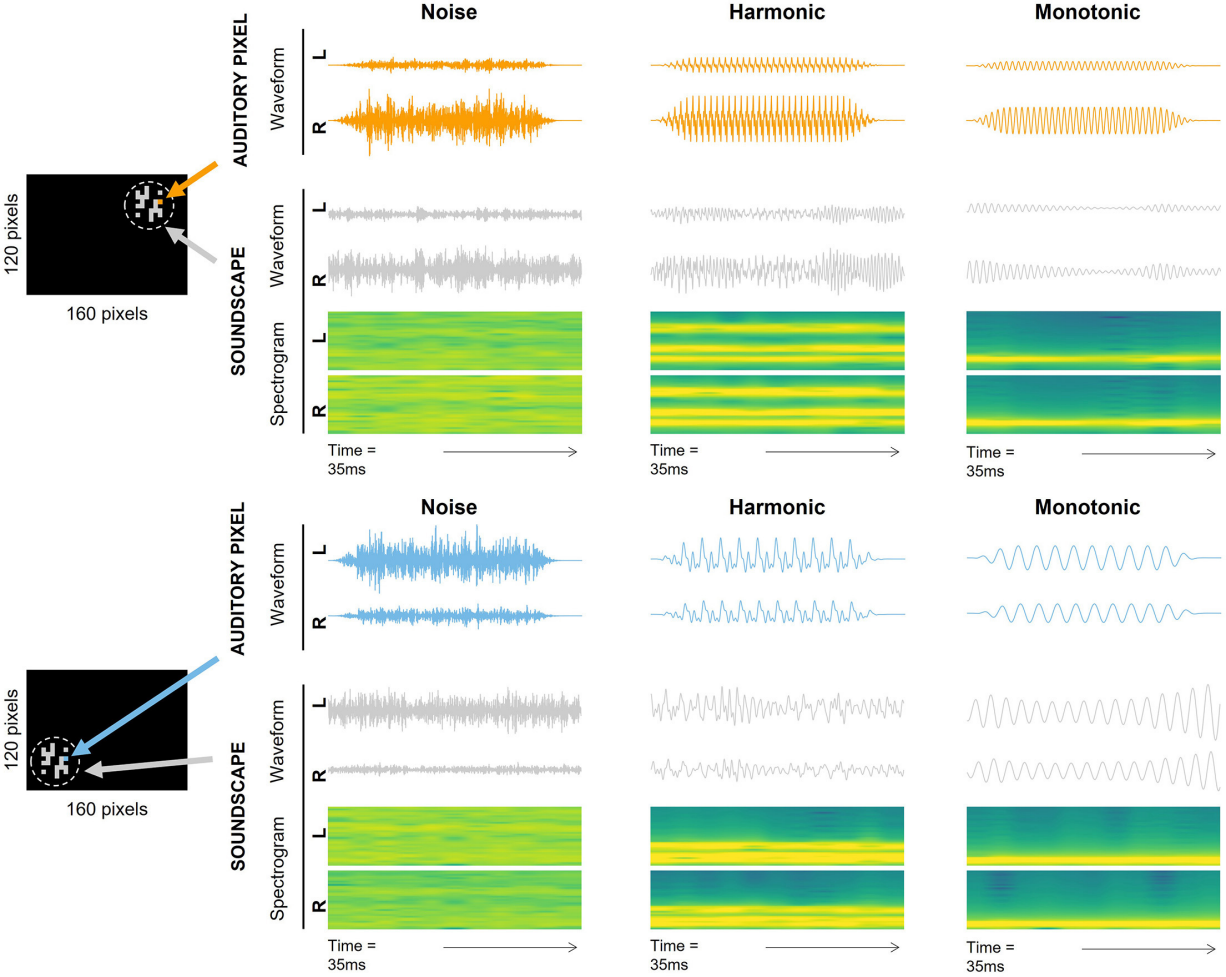
Two examples of processed video frames and their corresponding soundscapes. The two processed video frames are depicted in the left side of the figure with a target located on the upper right **(top image)** and bottom left **(bottom image)**. Active and inactive graphical pixels are depicted in gray and black, respectively. Two graphical pixels are highlighted in the video frames (orange in the **top image**, blue in the **bottom image**) and the corresponding auditory pixel waveforms are depicted in the right part of the figure in orange and blue. The corresponding soundscape waveforms (in gray) and soundscape spectrograms of the video frames are also depicted in the right part of the figure. Auditory pixel waveforms, soundscapes waveforms and spectrograms are displayed separately for the Noise encoding **(left column)**, the Harmonic encoding **(middle column)** and the Monotonic encoding **(right column)** and for left (L) and right (R) ear channels separately.

Video processing principles are similar to those used by Ambard et al. (2015), aiming to convey only new visual information from one frame to another. Video frames are grayscale images with gray levels ranging from 0 to 255. Pixels of the current frame are only conserved if the gray level pixel-by-pixel absolute difference with the previous frame (frame differencing) is larger than a threshold of 10. The processed image is then rescaled to a 160 × 120 (Horizontal × Vertical) grayscale image where 0-gray-level pixels are called “inactive” (i.e., no new visual information contained) and the others are “active” graphical pixels (i.e., containing new visual information). Active graphical pixels are then converted into spatialized sounds following a visual-to-auditory encoding in order to generate a soundscape (Figure 2), as explained in the following section.

#### 2.2.4. Visual-to-auditory conversion

The visual-to-auditory conversion consists in the transformation of the processed video stream into a synchronized audio stream that acoustically encodes the extracted graphical features. Each graphical pixel is associated with an “auditory pixel” which is a stereophonic sound with auditory cues specific to the position of the graphical pixel it is associated with. The conversion from a graphical pixel to an auditory pixel follows an encoding that is explained step-by-step in the following sections. Each graphical pixel of a video frame is first associated with a corresponding monophonic audio pixel (detailed in Section 2.2.4.1). The spatialization of the sound using HRTFs is then used to generate a stereophonic audio pixel that simulates a sound source with azimuth and elevation corresponding to the position of the graphical pixel in the virtual camera's field of view (detailed in Section 2.2.4.2). All the stereophonic pixels of a video frame are then compiled to obtain an audio frame (detailed in Section 2.2.4.3). Successive audio frames are then mixed together to generate a continuous audio stream (i.e., the soundscape). Two examples of stereophonic auditory pixels are provided in Figure 2 for each of the three encodings, as well as two examples of soundscapes depending on the location of an object in the field of view of the virtual camera.

##### 2.2.4.1. Monophonic pixel synthesizing

Three visual-to-auditory encodings were tested in this study: the Noise encoding and 2 Pitch encodings (the Monotonic encoding and the Harmonic encoding). These methods varied in the elevation encoding scheme and in the spectral complexity of the monophonic auditory pixels but all three methods used afterwards the same method for the sound spatialization.

For the Noise encoding, the simulated sound source (i.e., monophonic auditory pixel) in the VAS was a white noise signal generated by inverting a Fourier representation of the auditory pixel with a flat spectrum and random phases.

For the Monotonic encoding, each monophonic auditory pixel was a sinusoidal waveform audio signal (i.e., a pure tone) with a random phase and a frequency related to the elevation of the corresponding graphical pixel in the processed image. For this purpose, we used a linear Mel scale ranging from 344 mel (bottom) to 1,286 mel (top) corresponding to frequencies from 250 to 1,492 Hz.

For the Harmonic encoding, we used the same monophonic auditory pixels as in the Monotonic encoding but instead of a pure tone, we added to it two other frequencies at the 2 following octaves with the same intensity and random phases.

Since the loudness depends on the frequency components of the audio signal, we minimized the differences in loudness between auditory pixels using the *pyloudnorm* Python-package (Steinmetz and Reiss, 2021). Auditory pixel spectrums were then adjusted to compensate for the frequency response of the headphones we used in this experiment (SONY MDR-7506).

##### 2.2.4.2. Auditory pixel spatialization

The azimuth and elevation associated with each pixel were computed based on the coordinates of the corresponding graphical pixel in the camera's field of view. Monophonic auditory pixels were then spatialized by convolving them with the corresponding KEMAR HRTFs from the CIPIC database (Algazi et al., 2001a). This database provides HRTFs recordings with a sound source located in various azimuths and elevations ranging in steps of 5 and 5.625°, respectively. For each pixel, the applied HRTFs were estimated from the database by computing a 4 points time-domain interpolation in which the Interaural Level Difference (ILD) and the convolution signals were separately interpolated using bilinear interpolations before being reassembled as in Sodnik et al. (2005) but using a 2D interpolation instead of a 1D interpolation.

##### 2.2.4.3. Audio frame mixing

Each auditory pixel lasted 34.83 ms including a 5 ms cosine fade-in and a 5 ms cosine fade-out. All the auditory pixels corresponding to the active graphical pixels of the processed current video frame were compiled to form an audio frame. After their compilation, these fade-in and fade-out were still present at the beginning and at the end of the audio frame and they were used to overlap successive audio frames while limiting the artifacts of the auditory transition.

#### 2.2.5. Pointing tool and response collection

The pointing tool was a tracked gun pistol. Participants were instructed to indicate the perceived target position by pointing to it with the gun, with stretched arm. Participants logged their response by pressing a button with their index finger. They were instructed to hold the pointing tool with their dominant hand. The response position was defined as the intersection point of a virtual ray originating at the tip of the pointing tool and a virtual 1-m radius sphere with the origin at the location of the virtual camera. The response positions were declined in the elevation response and the azimuth response. The elevation and azimuth signed errors were also computed as the difference between the target position and the response position (in elevation and azimuth separately). A negative elevation signed error indicated a downward shift, and a negative azimuth signed error indicated a shift to the left in the response position. Unsigned errors were computed as the absolute value of the signed errors of each trial.

### 2.3. Experimental procedure

**Figure 3:**
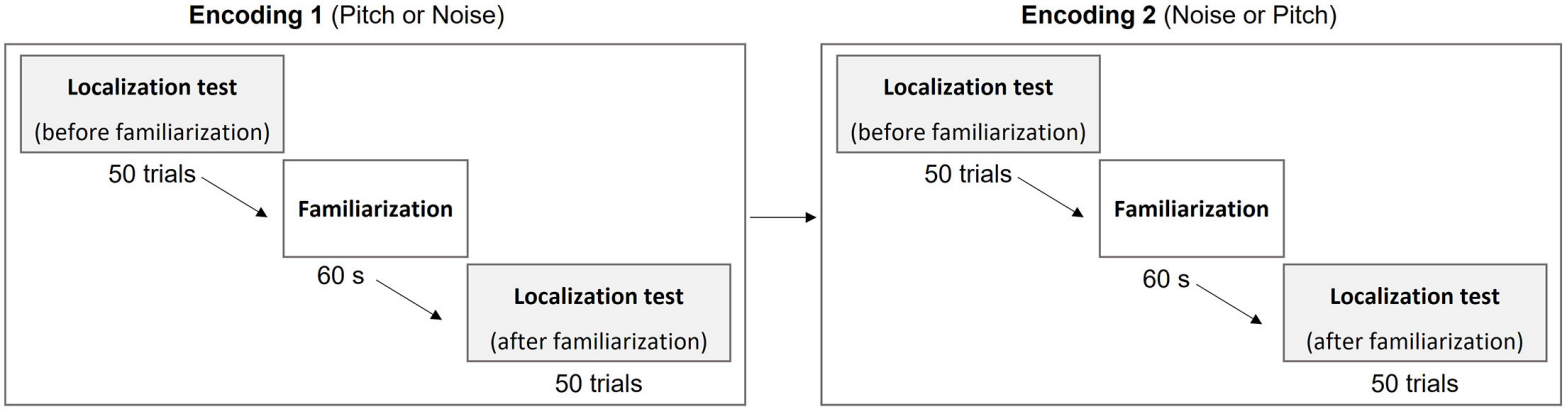
Experimental timeline. Participants had to sequentially test the Noise encoding and one of the two Pitch encodings (Monotonic or Harmonic). Participants of the Monotonic and Harmonic groups tested the Monotonic encoding and the Harmonic encoding respectively. For each encoding, participants practiced the localization test two times, before and after a familiarization session.

The experiment consisted in a 45-min session during which participants were seated comfortably in a chair at the center of a room surrounded by the virtual reality tracking system. The participants were equipped with SONY MDR-7506 headphones used to deliver soundscapes. Figure 3 illustrates the timeline of the experimental session. Each participant had to test two visual-to-auditory encodings: the Noise encoding, and a Pitch encoding (Monotonic or Harmonic encoding depending on the group they belonged). Participants from the Monotonic group had to test the Noise encoding and the Monotonic encoding, and participants from the Harmonic group had to test the Noise encoding and the Harmonic encoding. The order of the two tested encodings was counterbalanced between participants so half participants of each group started with the Noise encoding and the other half started with the Pitch encoding.

For each encoding, the participants practiced 2 times the localization test: one without any familiarization or explanation of the encoding and one after a familiarization session. At the beginning of the experiment, participants were instructed to localize a virtual target by pointing to it while being blindfolded. The experimenter explained that they will not be able to see the virtual target, but that they will only hear it and that the sound will depend on the position of the target. No indication was given about the way visual-to-auditory encodings worked. Participants were seated and blindfolded using an opaque blindfold fixed with a rubber band and could remove it during breaks. Participants were instructed to keep their head as still as possible during the localization tests. For control purposes, participants' head position was recorded with the tracker every 200 ms to check that they kept their head still. We measured the maximum distance of the head from its mean position for each trial and we found an average maximum distance of approximately 1.5 cm showing that the instructions were rigorously followed.

#### 2.3.1. Localization test

The localization test consisted in 50 trials during which blindfolded participants had to localize the virtual target using soundscapes provided by the visual-to-auditory SSD. During each localization test, the target was located at 25 different positions distributed on a grid of 5 azimuths (−40, −20, 0, +20, and +40°) and 5 elevations (−25, −12.5, 0, +12.5, and +25°). Figure 1B illustrates the grid with the 25 tested positions. As an example, the position [0°, 0°] corresponded to the central position, i.e., the virtual target was centered with the participant's head tracker. For the position [−40°, +12.5°], the target was 40° leftward and 12.5° upward from the central position ([0°, 0°]). The order of the tested positions was randomized and each position was tested 2 times per localization test. The target was placed at 1-meter-distance from the participant's head tracker for all positions (on the virtual 1-meter radius sphere used to collect the response positions).

Each trial started with a 500 ms 440 Hz beep sound, indicating the beginning of the trial. After a 500 ms silent period, the virtual target was displayed at one of the 25 tested positions. Participants were instructed to point with the pointing tool to the perceived location of the target with stretched arm. No time limit was imposed for responding but participants were asked to respond as fast and accurately as possible. The virtual target was displayed until participants pressed the trigger of the pointing tool. The response position was recorded (see Section 2.2.5 for response position computing) and the target disappeared. After a 1,000 ms inter-trial break, the next trial began with the 500 ms beep sound. No feedback was provided regarding response accuracy.

#### 2.3.2. Familiarization session

In between the 2 localization tests of each of the 2 tested encodings, participants practiced a familiarization session which consisted in a 60-s period during which participants freely moved the pointing tool in the front field. Figure 1C illustrates the familiarization session. The virtual target was continuously placed (i.e., no need to press the trigger) on a 1-meter radius sphere centered with the camera position, on the axis of the pointing tool. Consequently, when participants moved their arm, the target was continuously placed at the corresponding position on the 1-meter radius sphere and they could hear the soundscape provided by the encoding corresponding to the processed target images within the camera's field of view. The virtual camera position was updated one time at the beginning of the 60-s timer.

### 2.4. Data analysis

Statistical analysis were performed using R (version 3.6.1) (Team, 2020). Localization performance during localization tests was assessed separately for azimuth and elevation dimensions, with error-based and regression-based metrics, both fitted with Linear mixed models (LMMs) in order to take into account participants as random factor. All trials of all participants were included in the models without averaging the response positions or the unsigned errors by participant. The LMMs were fitted using the *lmerTest* R-package (Kuznetsova et al., 2017). We used an ANOVA with Satterthwaite approximation of degrees-of-freedom to estimate the effects. *Post-hoc* analysis were conducted using the *emmeans* R-package (version 1.7.4) (Lenth, 2022) with Tukey HSD correction.

#### 2.4.1. Error-based metrics with unsigned and signed errors

Localization performance was assessed through unsigned and signed errors. The elevation signed errors and azimuth signed errors were computed as the difference between target position and response position in each trial. A negative elevation signed error indicated a downward shift, and a negative azimuth signed error indicated a shift to the left in the response. Only descriptive statistics were conducted on the signed errors. The unsigned errors were computed as the absolute value of the signed error for each trial. They were investigated using LMMs including Encoding (Noise or Pitch), Group (Monotonic or Harmonic) and Phase (Before or After the familiarization) as fixed factors. Therefore, the positions of the target were not included as a factor in the LMMs of the unsigned error. Participants were considered as random effect in both models.

#### 2.4.2. Regression-based metrics with response positions

LMMs were also used for the analysis of the response positions. LMMs included Encoding (Noise or Pitch), Group (Monotonic or Harmonic), Phase (Before or After the familiarization), and Target position as fixed effects. The target elevation only, and the target azimuth only, were included in the elevation response LMM, and in the azimuth response LMM, respectively. Participants were considered as random effect in both models. We used the LMMs predictions to approximate the elevation and the azimuth gains and biases. The gains and biases were obtained by computing the trends (slopes) and intercepts of the models expressing the response position as a function of target position. Note that an optimal localization performance would be obtained with a gain value of 1.0 and a bias of 0.0°.

## 3. Results

### 3.1. Performance in elevation localization

**Figure 4:**
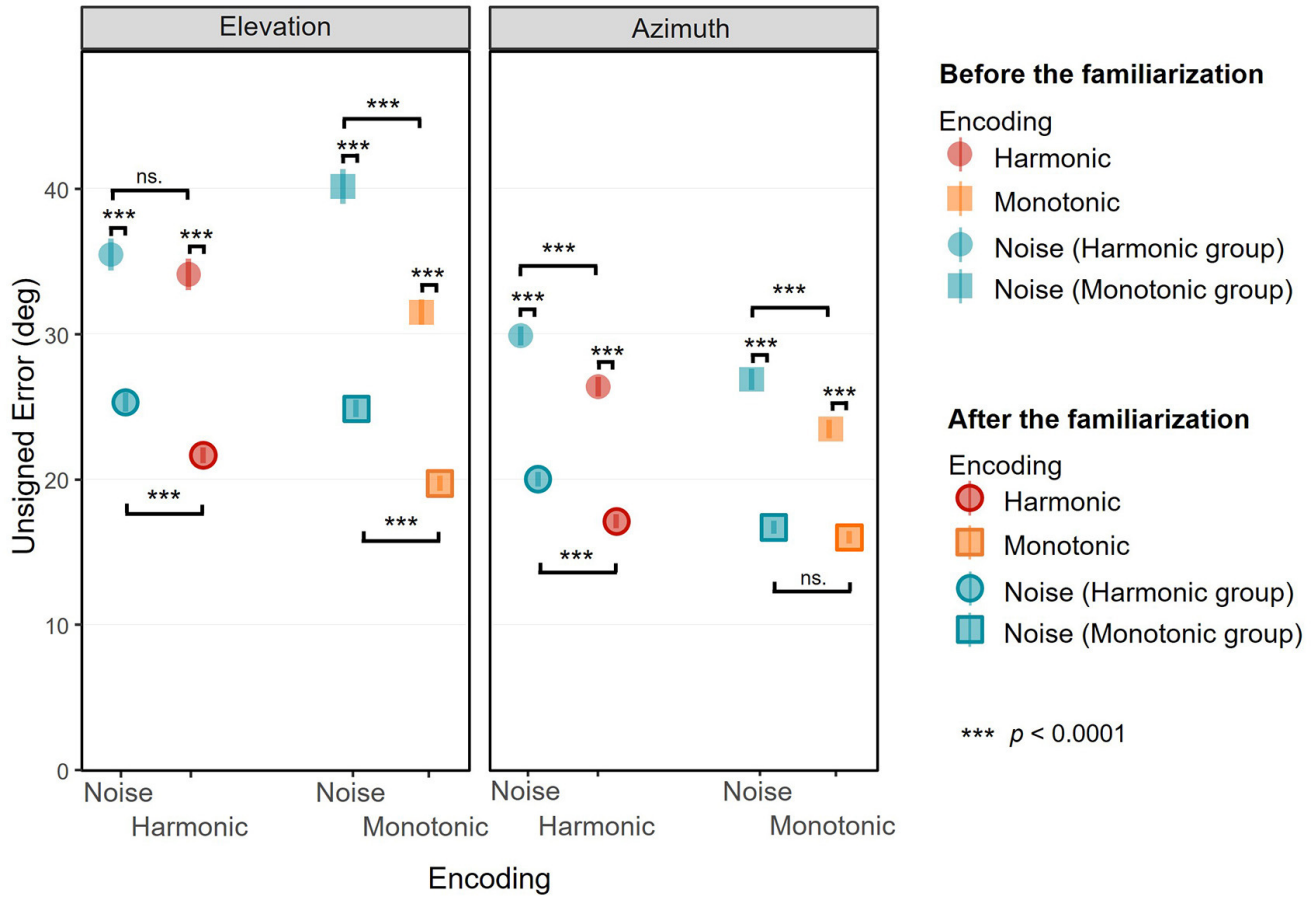
Unsigned error in elevation **(left)** and in azimuth **(right)** as a function of the encoding, all target positions combined. Mean unsigned errors (in degree) before (non-surrounded) and after (surrounded) are depicted separately for the Monotonic group (squares) and Harmonic group (circles) and for the three visual-to-auditory encodings: the Noise (blue), the Monotonic (orange) and the Harmonic (red) encodings. Error bars show standard error of the unsigned error.

The elevation unsigned errors are depicted in Figure 4, left, all target positions combined. Table 1 shows the elevation signed and unsigned errors for each Target elevation, Phase, Encoding and Group. The ANOVA on elevation unsigned errors showed a significant interaction effect of Phase × Encoding × Group [*F*_(1, 7556)_ = 6.23, *p* = 0.0126, $\eta_{p}^{2}$ = 0.0008]. *Post-hoc* analysis were conducted to investigate the interaction.

**Table 1:** Elevation signed error and unsigned error (in degree) for each encoding and target elevation, before, and after the familiarization session.

**Encoding**	**Target elevation** **(degree)**	**Elevation signed error (degree) Mean** ±**standard deviation**		**Elevation unsigned error (degree)** **Mean** ±**standard deviation**	
		**Before familiarization**	**After familiarization**	**Before familiarization**	**After familiarization**
Monotonic	+25	−26.71 ± 41.40	−13.21 ± 20.02	37.79 ± 31.55	18.55 ± 15.17
	+12.5	−28.09 ± 33.09	−16.55 ± 22.28	33.45 ± 27.62	21.99 ± 16.89
	0	−18.83 ± 36.41	−11.94 ± 22.73	31.63 ± 26.01	19.60 ± 16.55
	−12.5	−15.14 ± 34.95	−13.23 ± 24.95	27.30 ± 26.50	20.73 ± 19.14
	−25	−8.82 ± 34.34	−15.81 ± 15.18	27.51 ± 22.29	17.89 ± 21.65
Harmonic	+25	−17.66 ± 47.39	−13.73 ± 26.16	36.66 ± 34.76	23.03 ± 18.45
	+12.5	−7.18 ± 45.40	−6.61 ± 26.46	33.41 ± 31.47	20.73 ± 17.67
	0	−5.68 ± 45.71	−10.28 ± 28.07	32.91 ± 32.14	24.06 ± 17.67
	−12.5	−1.10 ± 48.75	−16.43 ± 21.80	33.44 ± 35.40	22.23 ± 15.81
	−25	2.99 ± 48.76	−15.85 ± 16.08	34.28 ± 34.72	18.44 ± 13.01
Noise (Monotonic group)	+25	−42.69 ± 52.91	−33.92 ± 27.00	56.49 ± 37.73	37.54 ± 21.64
	+12.5	−32.93 ± 45.45	−23.24 ± 23.54	43.63 ± 35.23	28.00 ± 17.56
	0	−25.49 ± 43.51	−12.98 ± 24.78	36.61 ± 34.62	23.09 ± 15.73
	−12.5	−20.61 ± 43.35	−7.09 ± 22.23	32.93 ± 34.88	19.02 ± 13.46
	−25	−8.40 ± 47.90	3.11 ± 22.32	31.30 ± 37.15	16.86 ± 14.90
Noise (Harmonic group)	+25	−34.74 ± 46.47	−33.67 ± 28.35	47.86 ± 32.71	36.96 ± 23.87
	+12.5	−24.89 ± 45.12	−21.25 ± 27.08	40.59 ± 31.66	27.87 ± 20.16
	0	−15.68 ± 42.71	−12.20 ± 25.02	32.41 ± 31.87	22.15 ± 16.81
	−12.5	−7.03 ± 44.28	−3.40 ± 25.66	28.84 ± 34.27	19.68 ± 16.76
	−25	0.69 ± 43.84	8.72 ± 25.46	27.84 ± 33.81	20.10 ± 17.85

**Figure 5:**
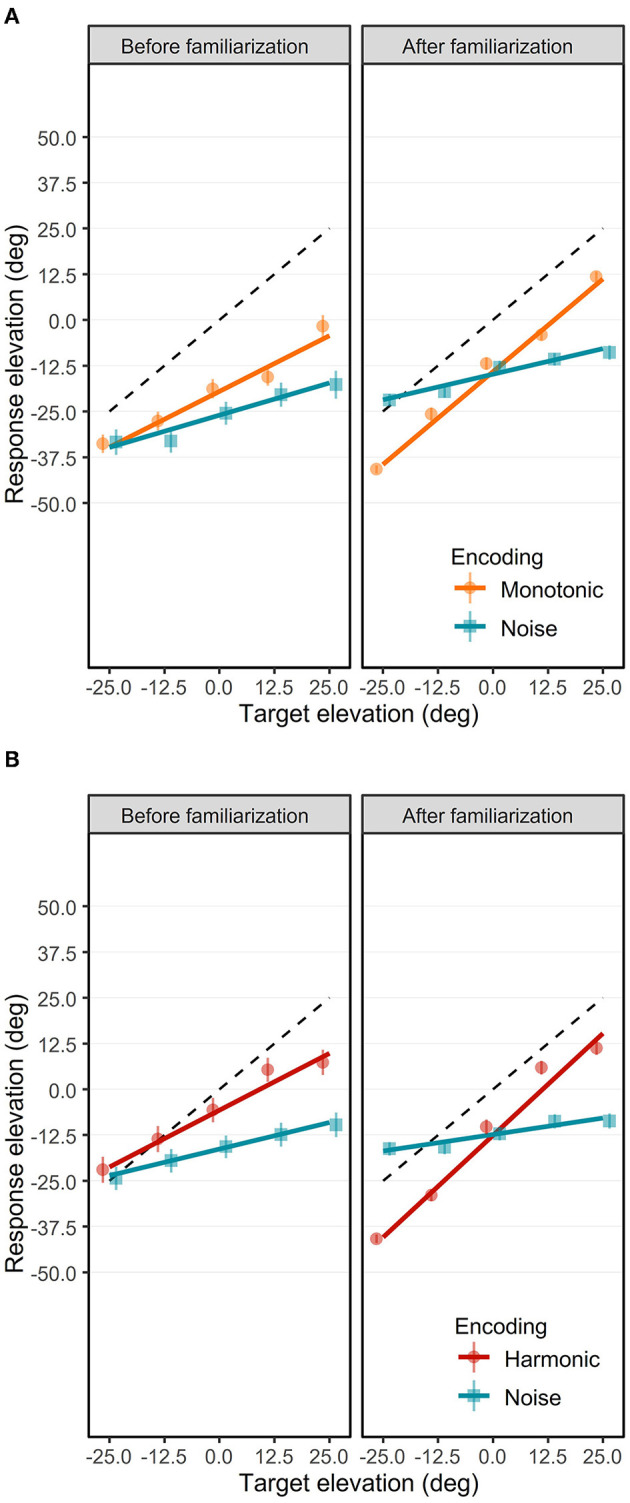
Elevation response position as a function of target elevation in the Monotonic group **(A)** and the Harmonic group **(B)**. Mean elevation response positions (in degree) before **(left)** and after **(right)** are represented separately for the three visual-to-auditory encodings: the Noise (blue squares), the Monotonic (orange circles) and the Harmonic (red circles) encodings. Error bars show standard error of elevation response position. Solid lines represent the elevation gains with the Noise (blue), the Monotonic (orange) and Harmonic (red) encodings. Black dashed lines indicate the optimal elevation gain 1.0.

The elevation response positions are depicted in Figure 5. The ANOVA showed a significant interaction effect of Phase × Target Elevation × Encoding [*F*_(1, 7548)_ = 38.84, *p* < 0.0001, $\eta_{p}^{2}$ = 0.005]. We conducted *post-hoc* analysis to investigate the elevation gain (the trend of the model) and bias (the intercept of the model) depending on the Phase and the Encoding. Although the interaction effect of Phase × Target Elevation × Encoding × Group was not significant [*F*_(1, 7548)_ = 0.50, *p* = 0.48, $\eta_{p}^{2}$ = 0.00007], *post-hoc* analysis were also performed for a control purpose in order to check for differences between the Monotonic and Harmonic groups. The elevation response positions are provided separately for each participant in the Supplementary Figures S1, S2.

#### 3.1.1. Elevation localization performance before the familiarization

Before the practice of the familiarization session, and depending on the encoding, the elevation unsigned errors were comprised between 31.54 ± 27.19° and 40.19 ± 37.02°. For the Monotonic group, the elevation unsigned errors were significantly lower with the Monotonic encoding (*M* = 31.54, *SD* = 27.19) than with the Noise encoding (*M* = 40.19, *SD* = 37.03) [*t*_(7556)_ = 7.457, *p* < 0.0001], suggesting a lower accuracy with the Noise encoding. There was no significant difference in the Harmonic group regarding the elevation unsigned error between the Harmonic encoding (*M* = 34.14, *SD* = 33.69) and the Noise encoding (*M* = 35.51, *SD* = 33.69).

The elevation response positions before the familiarization are depicted in the left panels of the Figures 5A, B for the Monotonic group and the Harmonic group, respectively. The elevation gains were significantly different from 0.0 for all encodings: 0.62 [95% CI = [0.5, 0.74], *t*_(7548)_ = 10.118, *p* < 0.0001] with the Harmonic encoding, 0.61 [95% CI = [0.49, 0.73], *t*_(7548)_ = 9.94, *p* < 0.0001] with the Monotonic encoding, and 0.29 [95% CI = [0.17, 0.41], *t*_(7548)_ = 4.728, *p* < 0.0001] and 0.35 [95% CI = [0.23, 0.47], *t*_(7548)_ = 5.746, *p* < 0.0001] with the Noise encoding of the Harmonic and Monotonic groups, respectively. It suggests that participants could discriminate different elevation positions with the three encodings even before the familiarization.

However, elevation gains were significantly lower than the optimal gain 1.0 with all encodings: with the Harmonic encoding [*t*_(7548)_ = −6.173, *p* < 0.0001], with the Monotonic encoding [*t*_(7548)_ = −6.351, *p* < 0.0001], and with the Noise encoding of the Harmonic group [*t*_(7548)_ = −11.562, *p* < 0.0001] and of the Monotonic group [*t*_(7548)_ = −10.544, *p* < 0.0001]. It depicts a situation where although some variations in elevation seemed to be perceived with the three encodings, participants had difficulties to estimate it before the familiarization.

The participants tended to localize the elevation with a higher performance with the Harmonic or Monotonic encoding than with the Noise encoding. Indeed, the participants from the Harmonic group showed a higher elevation gain with the Harmonic encoding than with the Noise encoding with a significant difference of 0.33 [*t*_(7548)_ = −3.811, *p*= 0.0008]. For the Monotonic group, the elevation gain was also significantly higher with the Monotonic encoding than with the Noise encoding with a difference of 0.26 [*t*_(7548)_ = −2.97, *p*= 0.016]. There was no significant difference regarding the elevation gain between the Harmonic and the Monotonic encodings.

The participants tended to underestimate the elevation position of the targets with the three encodings, as indicated by downward bias and negative elevation errors. In the Monotonic group, the elevation bias were −26.02° (95% CI = [−31.9, −20.16]) with the Noise encoding and −19.52° (95% CI = [−25.4, −13.65]) with the Monotonic encoding. In the Harmonic group, the elevation bias with the Noise encoding and with the Harmonic encoding were −16.33° (95% CI = [−22.2, −10.47]) and −5.73° (95% CI = [−11.6, 0.14]), respectively. With the exception of the Harmonic encoding for which there was just a trend [*t*_(44.9)_ = 1.97, *p*= 0.055], all the elevation bias mentioned above were significantly negative [all |*t*_(44.9)_| > 5.61, all *p* < 0.0001].

To sum up, participants appeared partially able to perceive a variation of the elevation position of the target with the three encodings before the audio-motor familiarization. Interestingly, participants seemed better able to localize the elevation with the Harmonic and Monotonic encodings.

#### 3.1.2. Elevation localization performance after the familiarization

After the familiarization, the elevation unsigned errors were significantly higher with the Noise encoding than with the 2 pitch-based encodings (Monotonic or Harmonic encodings). With the Noise encoding, the elevation unsigned errors were 24.90 ± 18.40° in the Monotonic group and 25.35 ± 20.31° in the Harmonic group. With the Harmonic and Monotonic encodings, the elevation unsigned errors were 21.70 ± 16.72° and 19.75 ± 16.25° respectively. In the Monotonic group, the elevation unsigned errors were significantly lower with the Monotonic encoding (*M* = 19.75, *SD* = 16.25) than with the Noise encoding (*M* = 24.90, *SD* = 18.40) [*t*_(7556)_ = 4.44, *p* < 0.0001]. Unlike before the familiarization, the difference was also significant in the Harmonic group. The elevation unsigned errors with the Harmonic encoding (*M* = 21.70, *SD* = 16.72) were lower than with the Noise encoding (*M* = 25.35, *SD* = 20.31), [*t*_(7556)_ = 3.15, *p*= 0.0016]. Interestingly, the elevation unsigned errors significantly decreased after the familiarization with all the encodings [all |*t*_(7556)_| > 8.75, all *p* < 0.0001], suggesting that participants localized more accurately the elevation after the familiarization.

The elevation response positions after the familiarization are depicted in the Figures 5A, B for the Monotonic and Harmonic groups, respectively. After the familiarization, the elevation gains were still significantly higher than 0.0 [all |*t*_(7548)_| > 2.9152, all *p* < 0.0036] with all encodings in the 2 groups. The elevation gains were 1.112 (95% CI = [0.99, 1.23]) with the Harmonic encoding and 1.015 (95% CI = [0.89, 1.14]) with the Monotonic encoding. For the participants of the Harmonic group and the Monotonic group, the elevation gains with the Noise encoding were 0.179 (95% CI = [0.06, 0.30]), and 0.278 (95% CI = [0.16, 0.40]), respectively.

The elevation gains were significantly higher with the Harmonic and Monotonic encodings than with the Noise encoding. We measured a difference of 0.74 [*t*_(7548)_ = −8.49, *p* < 0.0001] in the Harmonic group and a difference of 0.93 [*t*_(7548)_ = −10.75, *p* < 0.0001] in the Monotonic group. Inter-group analysis showed that the difference in elevation gain between the Monotonic and the Harmonic encodings did not significantly differ [*t*_(7548)_ = 1.12, *p*= 0.95].

The elevation gains with the Harmonic and the Monotonic encodings significantly improved after the familiarization to get closer than the optimal gain 1.0. With the Harmonic encoding, the elevation gain significantly increased from 0.62 to 1.112 [*t*_(7548)_ = 5.66, *p* < 0.0001] after which it was not significantly different from the optimal gain 1.0 [*t*_(7548)_ = 1.832, *p*= 0.067]. With the Monotonic encoding, the elevation gain significantly increased from 0.61 to 1.015 [*t*_(7548)_ = 4.665, *p* < 0.0001], and was also no more significantly different from the optimal gain 1.0 [*t*_(7548)_ = 0.246, *p*= 0.806]. However with the Noise encoding in both groups, the familiarization did not improve the elevation gains. In the Harmonic and Monotonic groups, the elevation gains decreased from 0.29 to 0.179 and from 0.35 to 0.278, respectively, but, as previously reported, the decreases were not significant.

Participants kept tending to underestimate the elevation position of the targets with all three encodings, as indicated by persistent negative bias. In the Monotonic group, the elevation bias with the Noise encoding and with the Monotonic encoding were −14.82° (95% CI = [−20.7, −8.96]) and −14.15° (95% CI = [−20.0, −8.28]), respectively. In the Harmonic group, the elevation bias with the Noise encoding and with the Harmonic encoding were −12.36° (95% CI = [−18.2, −6.49]) and −12.58° (95% CI = [−18.4, −6.72]), respectively. All the elevation bias were significantly negative [all |*t*_(44.9)_| > 4.24, all *p* < 0.0001].

To sum up, after the familiarization, the perception of elevation with the Harmonic and Monotonic encodings improved with elevation gains getting closer to the optimal gain. However, the familiarization did not induce any significant improvement in the perception of elevation with the Noise encoding, with persistent low elevation gains in both groups. Additionally, the underestimation elevation bias decreased with the Monotonic and Noise encodings, but not with the Harmonic encoding for which it increased.

### 3.2. Performance in azimuth localization

The azimuth unsigned errors are depicted in Figure 4, right, all target positions combined. Table 2 shows the azimuth signed and unsigned errors for each Target azimuth, Phase, Encoding and Group. The ANOVA on azimuth unsigned errors showed a significant interaction effect of Phase × Encoding [*F*_(1, 7556)_ = 5.15, *p*= 0.023, $\eta_{p}^{2}$ = 0.00068], but the interaction including the group was not significant.

**Table 2:** Azimuth signed error and unsigned error (in degree) for each encoding and target azimuth, before, and after the familiarization session.

**Encoding**	**Target azimuth (degree)**	**Azimuth signed error (degree) Mean** ±**standard deviation**		**Azimuth unsigned error (degree)** **Mean** ±**standard deviation**	
		**Before familiarization**	**After familiarization**	**Before familiarization**	**After familiarization**
Monotonic	+40	17.79 ± 23.73	4.39 ± 18.25	23.16 ± 18.5	13.97 ± 12.5
	+20	25.34 ± 25.50	12.06 ± 18.35	28.71 ± 21.61	17.14 ± 13.69
	0	−7.75 ± 25.39	−5.02 ± 19.33	17.95 ± 19.52	15.21 ± 12.90
	−20	−25.25 ± 21.81	−15.02 ± 18.10	26.93 ± 19.69	18.68 ± 14.27
	−40	−16.27 ± 20.33	−5.34 ± 19.40	20.68 ± 15.79	15.23 ± 13.11
Harmonic	+40	23.26 ± 19.59	5.43 ± 21.49	24.9 ± 17.45	15.83 ± 15.48
	+20	27.17 ± 20.26	12.98 ± 18.80	28.17 ± 19.61	17.23 ± 14.98
	0	−6.89 ± 23.03	−6.02 ± 18.49	16.89 ± 17.36	14.15 ± 12.97
	−20	−32.76 ± 23.38	−16.61 ± 19.93	33.16 ± 22.81	21.6 ± 14.35
	−40	−27.45 ± 23.93	−7.03 ± 22.08	29.25 ± 21.68	16.68 ± 16.05
Noise (Monotonic group)	+40	23.01 ± 25.34	7.27 ± 16.58	27.91 ± 19.79	14.35 ± 11.01
	+20	28.35 ± 29.17	11.96 ± 19.59	30.95 ± 26.38	18.11 ± 14.06
	0	−8.89 ± 24.82	−8.47 ± 14.79	16.78 ± 20.31	12.04 ± 12.05
	−20	−31.27 ± 25.61	−21.37 ± 17.24	33.42 ± 22.71	22.32 ± 15.98
	−40	−21.22 ± 24.10	−11.55 ± 16.88	25.53 ± 19.45	16.82 ± 11.59
Noise (Harmonic group)	+40	24.13 ± 23.85	9.86 ± 24.77	28.65 ± 18.12	19.17 ± 18.49
	+20	29.80 ± 24.77	15.02 ± 17.67	31.78 ± 22.16	18.94 ± 13.35
	0	−11.84 ± 27.02	−7.04 ± 20.76	21.10 ± 20.57	16.34 ± 14.57
	−20	−36.95 ± 20.92	−22.36 ± 18.41	37.28 ± 20.31	25.06 ± 14.48
	−40	−30.29 ± 17.83	−12.40 ± 23.27	30.71 ± 17.09	20.71 ± 16.28

**Figure 6:**
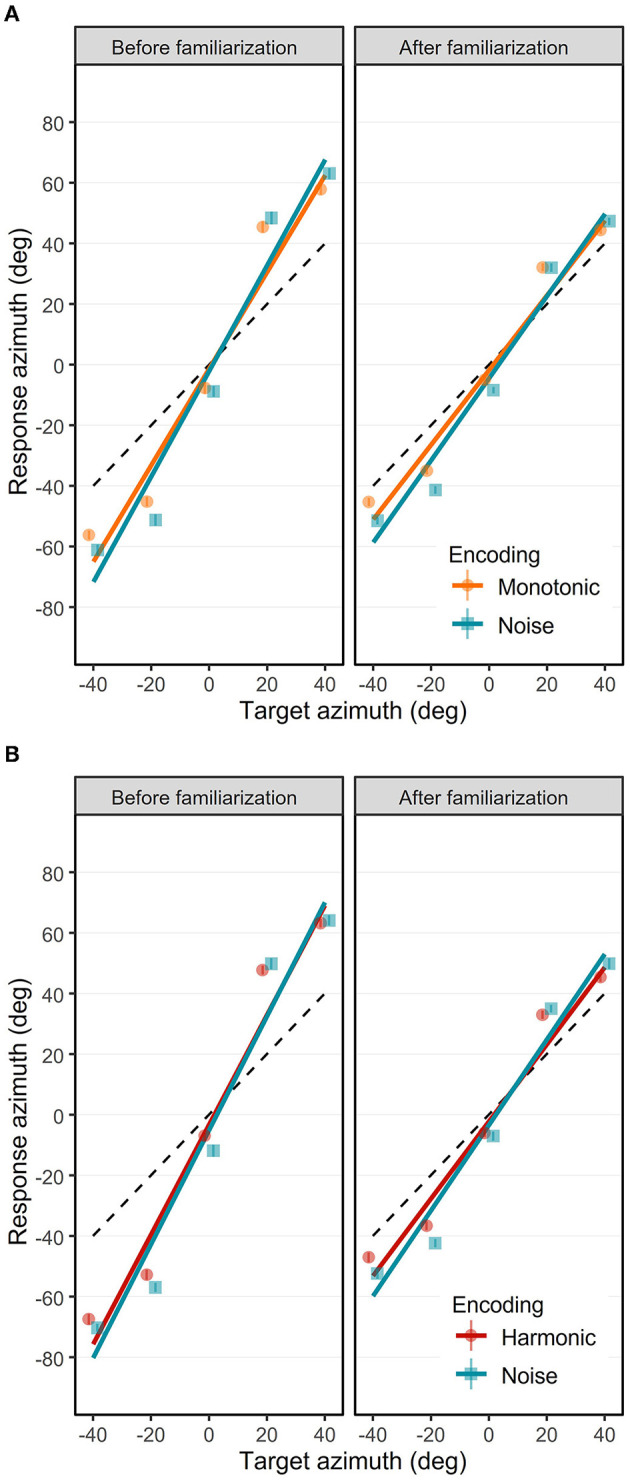
Azimuth response position as a function of target azimuth in the Monotonic group **(A)** and the Harmonic group **(B)**. Mean azimuth response positions (in degree) before **(left)** and after **(right)** are represented separately for the three visual-to-auditory encodings: the Noise (blue squares), the Monotonic (orange circles) and the Harmonic (red circles) encodings. Error bars show standard error of azimuth response position. Solid lines represent the azimuth gains with the Noise (blue), the Monotonic (orange) and Harmonic (red) encodings. Black dashed lines indicate the optimal azimuth gain 1.0.

The azimuth response positions are depicted in Figure 6. The ANOVA yielded a significant interaction effect of Phase × Target Azimuth × Encoding [*F*_(1, 7548)_ = 12.69, *p*= 0.0004, $\eta_{p}^{2}$ = 0.00005]. *Post-hoc* analysis were conducted to investigate the azimuth gain (the trend of the model) and bias (the intercept of the model) depending on the Phase and the Encoding. Although the interaction effect of Phase × Target Elevation × Encoding × Group was not significant [*F*_(1, 7548)_ = 1.64, *p* = 0.20, $\eta_{p}^{2}$ = 0.0002], we conducted *post-hoc* analysis to check for differences between the Monotonic and Harmonic groups for a control purpose. The azimuth response positions are provided separately for each participant in the Supplementary Figures S3, S4.

#### 3.2.1. Azimuth localization performance before the familiarization

Before the practice of the familiarization session, and depending on the encoding, the azimuth unsigned errors were comprised between 23.48 ± 19.48° and 29.91 ± 20.38°. In the Monotonic group, the azimuth unsigned errors were significantly lower with the Monotonic encoding (*M* = 23.48, *SD* = 19.48) than with the Noise encoding (*M* = 26.92, *SD* = 22.58) [*t*_(7556)_ = 4.64, *p* < 0.0001]. The azimuth unsigned errors in the Harmonic group were also significantly lower [*t*_(7556)_ = 4.73, *p* < 0.0001] with the Harmonic encoding (*M* = 26.41, *SD* = 20.63) than with the Noise encoding (*M* = 29.91, *SD* = 33.69).

The azimuth response positions over all participants before the familiarization are depicted in the left panels of the Figures 6A, B for the Monotonic and Harmonic groups, respectively. Before the familiarization, the participants were able to interpret soundscapes to localize the target azimuth. First, the participants perceived different azimuth positions. Indeed, azimuth gains were significantly different from 0.0 with all encodings: 1.81 (95% CI = [1.75, 1.87], *t*_(7548)_ = 70.397, *p* < 0.0001) with the Harmonic encoding, 1.59 [95% CI = [1.54, 1.65], *t*_(7548)_ = 61.996, *p* < 0.0001] with the Monotonic encoding, and 1.88 [95% CI = [1.82, 1.93], *t*_(7548)_ = 73.0624, *p* < 0.0001] and 1.74 [95% CI = [1.68, 1.80], *t*_(7548)_ = 67.710, *p* < 0.0001] with the Noise encoding for the participants in the Harmonic and Monotonic groups, respectively.

Interestingly, the azimuth gains were significantly higher than the optimal gain (i.e., higher than 1.0) with the Harmonic encoding [*t*_(7548)_ = 31.492, *p* < 0.0001], the Monotonic encoding [*t*_(7548)_ = 23.091, *p* < 0.0001], and the Noise encoding in the Harmonic group [*t*_(7548)_ = 34.156, *p* < 0.0001] and the Monotonic group [*t*_(7548)_ = 28.804, *p* < 0.0001]. These gains higher than the optimal gain reflect a lateral overestimation (i.e., left targets localized too much on the left and right targets localized too much on the right) that can be seen with the three encodings.

In the Monotonic group, the overestimation observed with the Noise encoding was significantly higher than with the Monotonic encoding [*t*_(7548)_ = 4.04, *p*= 0.0003]. In the Harmonic group, the overestimation with the Noise encoding compared to the Harmonic encoding was also higher but not significantly. Inter-group comparison of the azimuth gains obtained with the Noise encoding shows a small but significant higher azimuth gain in the Harmonic group [difference of 0.14: *t*_(7548)_ = 3.784, *p*= 0.0009]. As inter-group comparison, we also observed a slight but significant higher overestimation pattern with the Harmonic encoding in comparison with the Monotonic encoding [difference of 0.22: *t*_(7548)_ = 5.94, *p* < 0.0001].

Another interesting result is the tendency to show a left shift as indicated by negative azimuth bias with the three encodings. With the Noise encoding in the Harmonic group, the leftward azimuth bias of −5.03° was significant [*t*_(47.2)_ = 2.84, *p*= 0.0066]. However, leftward azimuth bias with the other encodings were not significantly different from 0.0° (Harmonic group, Noise encoding: −3.23°; Monotonic group, Noise encoding: −2.0°; Monotonic group, Monotonic encoding: −1.233°).

In summary, before the familiarization and with the three encodings, participants were able to localize the azimuth of the target accurately with a tendency to overestimate the lateral eccentricity and a tendency to point too much on the left.

#### 3.2.2. Azimuth localization performance after the familiarization

After the participants practiced the familiarization session, and depending on the encoding, the azimuth unsigned errors were comprised between 16.05 ± 13.38° and 20.05 ± 15.77°. In the Harmonic group, the azimuth unsigned errors were significantly lower with the Harmonic encoding (*M* = 17.16, *SD* = 14.97) than with the Noise encoding (*M* = 20.05, *SD* = 15.77) [*t*_(7556)_ = 3.91, *p*= 0.0001]. The azimuth unsigned errors were not significantly different anymore between the Monotonic encoding (*M* = 16.05, *SD* = 13.38) and the Noise encoding (*M* = 16.73, *SD* = 13.50). Importantly, the azimuth unsigned errors significantly decreased after the familiarization session for all three encodings [all |*t*_(7556)_| > 10.06, all *p* < 0.0001], suggesting that participants localized more accurately the azimuth after the familiarization.

The azimuth response positions after the familiarization are depicted in the right panels of the Figures 6A, B for the Monotonic and Harmonic groups, respectively. As expected, after the familiarization, participants were still able to localize different azimuth positions by interpreting soundscapes. Azimuth gains were still significantly different from 0.0 with the Harmonic encoding [1.27, 95% CI = [1.22, 1.32], *t*_(7548)_ = 49.51, *p* < 0.0001], with the Monotonic encoding [1.23, 95% CI = [1.18, 1.28], *t*_(7548)_ = 47.96, *p* < 0.0001], and with the Noise encoding for the participants in the Harmonic group [1.41, 95% CI = [1.36, 1.46], *t*_(7548)_ = 54.84, *p* < 0.0001] and in the Monotonic group [1.35, 95% CI = [1.30, 1.41], *t*_(7548)_ = 52.711, *p* < 0.0001], respectively.

The overestimation pattern was still present, as indicated by azimuth gains still significantly higher than the optimal gain 1.0 with all encodings: the Harmonic encoding [*t*_(7548)_ = 10.61, *p* < 0.0001], the Monotonic encoding [*t*_(7548)_ = 9.06, *p* < 0.0001], the Noise encoding in the Harmonic group [*t*_(7548)_ = 15.93, *p* < 0.0001] and in the Monotonic group [*t*_(7548)_ = 13.81, *p* < 0.0001].

Although the lateral overestimation was still significant, it significantly decreased compared to the same localization test before the familiarization. Indeed, the azimuth gains decreased and reached values closer than the optimal gain 1.0 with the 3 encodings. There were significant decreases in azimuth gains of a magnitude of 0.54 [*t*_(7548)_ = 14.77, *p* < 0.0001] and 0.36 [*t*_(7548)_ = 9.92, *p* < 0.0001] with the Harmonic and Monotonic encodings, respectively. The decreases in azimuth gains with the Noise encoding in the Harmonic and the Monotonic groups were also significant with a decrease of a magnitude of, respectively, 0.47 [*t*_(7548)_ = 12.897, *p* < 0.0001] and 0.39 [*t*_(7548)_ = 10.61, *p* < 0.0001].

Additionally, after the familiarization, participants tended to localize the azimuth with a higher performance with the Harmonic and Monotonic encodings than with the Noise encoding. This is suggested by a more pronounced lateral overestimation with the Noise encoding in both groups: the azimuth gains were 0.14 higher [*t*_(7548)_ = 3.76, *p*= 0.0042] and 0.12 higher [*t*_(7548)_ = 3.36, *p*= 0.018] with the Noise encoding in comparison with the Harmonic and Monotonic encodings, respectively.

The slight tendency to show a left shift bias in azimuth was still present with the three encodings. With the Noise encoding in the Monotonic group, the leftward azimuth bias of −4.43° was significant [*t*_(47.2)_ = 2.502, *p*= 0.0159], but in the Harmonic group the bias of −3.38° was just a tendency [*t*_(47.2)_ = 1.911, *p*= 0.0621]. The leftward azimuth bias with the Harmonic encoding (−2.25°) and Monotonic encoding (−1.79°) were also not significant.

To sum up the accuracy in azimuth localization, participants were able to localize target azimuths accurately even before the audio-motor familiarization. After the familiarization, the accuracy increased with a decrease in both the tendency to overestimate the lateral position of lateral targets and the tendency to point too much on the left.

## 4. Discussion

In this study, we investigated the early stage of use of visual-to-auditory SSDs based on the creation of a VAS (Virtual Acoustic Space) for object localization in a virtual environment. Based on soundscapes created using non-individualized HRTFs, we investigated blindfolded participants' abilities to localize a virtual target with three encoding schemes: one conveying elevation with spatialization only (Noise encoding), and two conveying elevation with spatialization and pitch modulation (Monotonic and Harmonic encodings). The two pitch-based encodings varied regarding the sound spectrum complexity: one narrowband with monotones (Monotonic encoding) and one more complex with 2 additional octaves (Harmonic encoding). In order to compare the localization abilities for the azimuth and the elevation with the different visual-to-auditory encodings, we collected the response positions and angular errors of the participants during a task consisting in the localization of a virtual target placed at different azimuths and elevations in their front-field.

### 4.1. Elevation localization abilities using the visual-to-auditory encodings

#### 4.1.1. Elevation localization performance only based on non-individualized HRTFs is impaired

With the spatialization-based only encoding (Noise encoding), the target was localized before the familiarization with an elevation unsigned error between 27.84 ± 33.81° and 56.49 ± 37.73°. After the familiarization, the elevation unsigned errors decreased to reach values comprised between 16.86 ± 14.90° and 37.54 ± 21.64°. As a comparison, in Mendonça et al. (2013) where the same HRTFs database was used with a white noise sound, the mean elevation unsigned error of participants was 29.3° before practicing a training. The elevation unsigned errors in Geronazzo et al. (2018) without any familiarization and with a white noise sound were comprised between 15.58 ± 12.47° and 33.75 ± 16.17° depending on participants, which is comparable to our results after the familiarization. However, as shown by elevation gains below 0.4 before or after familiarization, the participants had difficulties to discriminate different elevations with this encoding.

The abilities to localize the elevation of an artificially spatialized sound are known to be impaired in comparison with azimuth (Wenzel et al., 1993). Those difficulties arise from the spectral distortions that are specific to individual body morphology (Blauert, 1996; Xu et al., 2007). When using non-individualized HRTFs, these spectral distortions are different from the participant's specific distortions, causing misinterpretation of elevation location. Additionally, the abilities to localize the elevation position of a sound source (virtual or real) are modulated by the spectral content of the sound (Middlebrooks and Green, 1991; Blauert, 1996).

In our study, the difficulty with the spatialization-based only encoding to localize the elevation of the target, even after the audio-motor familiarization, could be explained by a too brief training period to get used to the new auditory cues. Actually, some studies showed an improvement of localization abilities with non-individualized or modified HRTFs after 3 weeks of training in Majdak et al. (2013) or Romigh et al. (2017), or after 2 weeks in Shinn-Cunningham et al. (1998) or 1 week in Kumpik et al. (2010), and about 5 h in Bauer et al. (1966). Moreover, Mendonça et al. (2013) showed the positive long term effect (1-month long) of training in azimuth and elevation localization abilities with a sound source spatialized using the same HRTFs database that was used in the current study. It suggests that the exclusive use of HRTFs to encode spatial information in SSDs might require a long training period or a long process to acquire individualized HRTFs.

#### 4.1.2. Positive effects of cross-modal correspondence on elevation localization

The participants' abilities to localize the elevation of the target using the 2 pitch-based encodings were significantly better than with a broadband sound spatialization encoding. Before the audio-motor familiarization, with the narrowband encoding (Monotonic) and the more complex encoding (Harmonic), the unsigned errors in elevation were comprised between 27.30 ± 26.50° and 37.79 ± 31.55° depending on the target elevation.

Before the familiarization, participants did not receive any information about the way the sound was modulated depending on the target location. In other words, they did not know that low pitch sounds were associated with low elevation locations, and conversely. However, the individual results of each participant for the elevation (Supplementary Figures S1, S2) suggest that even before the familiarization, several participants interpreted the pitch to perceive the target elevation, using high pitch for high elevation and low pitch for low elevation. We suppose that participants were able to guess that the pitch of the sound varied with the target elevation because the experimenter explicitly told them that sound features were modulated as a function of the location of the target although no details regarding this modulation were provided. Two participants (S12 from the Harmonic group and S15 from the Monotonic group) reversed the pitch encoding by associating a low pitch to high elevations and a high pitch to low elevations, but they reversed this miss-representation after the familiarization. Our study showed that after the audio-motor familiarization, the elevation unsigned errors significantly decreased with both pitch-based encodings to reach values comprised between 17.67 ± 22.23° and 24.06 ± 17.67°, which are lower elevation unsigned errors than the mean elevation error of 25.2° immediately after the training in Mendonça et al. (2013).

In the visual-to-auditory SSD domain, the artificial pitch mapping of elevation is used by several existing visual-to-auditory SSDs and relies on the audiovisual cross-modal correspondence between visual elevation and pitch (Spence, 2011; Deroy et al., 2018). In the current study, the frequency range was between 250 Hz and about 1,500 Hz with the Monotonic encoding and between 250 Hz and about 6,000 Hz with the Harmonic encoding (i.e., 1,500 Hz × 2 × 2). The floor value of 250 Hz was chosen to provide frequency steps of at least 3 Hz between each of the 120 auditory pixels in a column, to fit to the human frequency discrimination abilities (Howard and Angus, 2009). We used the Mel scale (Stevens et al., 1937) to take into account the perceived scaling in sound frequency discrimination. All the SSDs using a pitch mapping of elevation use different frequency ranges, resolutions (i.e., number of used frequencies) and frequency steps. The vOICe SSD (Meijer, 1992) uses a larger frequency range than the current study (from 500 to 5,000 Hz) following an exponential scale with a 64-frequency resolution. The EyeMusic SSD (Abboud et al., 2014) uses a pentatonic musical scale with 24 frequencies from 65.785 Hz to 1577.065 Hz. The SSD proposed in Ambard et al. (2015) also uses 120 frequency steps but following the Bark scale (Zwicker, 1961) and with a larger frequency range (from 250 Hz to about 2,500 Hz). Technically, increasing the range of frequencies might increase discrimination abilities between target elevations and improve localization abilities. Although, as sound frequency increases the sound feels unpleasant (Kumar et al., 2008). We can postulate that SSD users should be able to modulate some of the parameters in order to adapt the encoding scheme to their own auditory abilities and perceptual preferences.

Our results suggest that a pitch mapping of elevation can quickly be interpreted, even without any explicit explanation of the mapping rules. They also suggest that the pitch mapping provides acoustic cues that are easily interpretable at the early stage of use of a SSD to localize an object. In terms of spatial perception, our study shows that adding abstract acoustic cues to convey spatial information can be more efficient than an imperfect synthesizing of natural acoustic cues. It is difficult to assert that the differences in the results between the Noise encoding and the Pitch encodings are entirely due to the cross-modal correspondence between elevation and pitch since modifying the timbre of the sound by reducing its spectral content also modified how the HRTFs spatialize the sound. Therefore, it would be interesting to investigate the localization performance with monotonic or harmonic sounds in which the pitch is constant (i.e., not related to the elevation of the target) and by conducting an experiment where HRTFs convolution is computed to convey azimuth only, with for instance a constant elevation of 0°.

#### 4.1.3. Insights about the pitch-elevation cross-modal correspondence

Although the aim of this study was not to directly investigate the multisensory perceptual process, the results might bring insights about the pitch-elevation cross-modal correspondence. In the SSD research, it has been suggested that the pitch-based elevation mapping is intuitive in an object recognition task (Stiles and Shimojo, 2015). Based on the results of the current study, it also seems intuitive in a localization task. However, it remains to be further investigated with, for instance, a comparison of elevation localization abilities with a similar pitch-based elevation encoding and another encoding where the direction of the pitch mapping is reversed (i.e., low pitch for high elevation and high pitch for low elevation). The current study also raises the question regarding the automaticity of the cross-modal correspondences as disccussed in Spence and Deroy (2013). In the current study, the facilitation effect of the cross-modal correspondence probably relies on voluntary multisensory perceptual processes. The way the instructions were given to the participants intrinsically induced a goal-directed voluntary strategy in order to infer which modifications in the sound could convey information about the location of the object.

These insights about multisensory process should also be investigated in the blind. Since the pitch-elevation cross-modal correspondence has been suggested to be weak in this population (Deroy et al., 2016), and since auditory spatial perception of the elevation can be impaired in this population (Voss, 2016), it remains to investigate whether similar results would be obtained with blind participants. For this reason, the procedure of the current study was designed in a way to be reproducible with blind participants.

#### 4.1.4. No positive effects of harmonics on elevation localization

The elevation-pitch encoding adds a salient auditory cue while reducing the frequency range where the HRTFs spectrum alterations can operate. To study the effect of the spectral complexity we used an encoding with harmonic sounds (monotonic and 2 following octaves) meant to be a trade-off in terms of spectral complexity between the broadband sound of the Noise encoding and the monotones of the Monotonic encoding. Although pure tones were used in the Monotonic encoding, it is important to keep in mind that soundscapes were not pure tones. Indeed, soundscapes were made of adjacent auditory pixels, resulting in narrowband but multi-frequency soundscapes (see Figure 2).

The results did not show inter-group differences in the localization accuracy between the Monotonic and the Harmonic encodings. It suggests that adding 2 octaves to the original sound (i.e., the Monotonic encoding) did not modulate the ability to perceive the elevation of the target. Using more complex tones with several sub-octave intervals in the Harmonic encoding might sufficiently modify the sound spectrum to obtain a significant difference with the Monotonic encoding. It could also be interesting to investigate the ability to perceive the elevation of the target with an encoding using sounds containing frequencies higher than the current ceiling frequency (6,000 Hz). However, as mentioned in Section 4.1.1, it seems that the benefits that could arise from the application of the HRTFs on a sound with a broader spectrum could only be perceivable after a long training period.

### 4.2. Azimuth localization using the visual-to-auditory encodings is accurate but overestimated

Depending on the encoding and the target eccentricity, the magnitude of the azimuth unsigned errors was comprised between 16.78 ± 20.31° and 37.29 ± 20.32°. As a comparison, Mendonça et al. (2013) spatialized white noise sounds using the same HRTFs database and their participants localized the azimuth with a mean unsigned error of 21.3° before the training practice. In Geronazzo et al. (2018), the azimuth unsigned errors of participants varied between 3.67 ± 2.97° and 35.98 ± 45.32°. In the SSD domain, Scalvini et al. (2022) found a mean azimuth error of 6.72 ± 5.82° in a task consisting in localizing a target with the head. In the current study, after the familiarization, the magnitude of azimuth unsigned errors decreased and was comprised between 12.04 ± 12.05° and 25.06 ± 14.48° depending on the azimuth eccentricity which is comparable to the azimuth unsigned errors found in Geronazzo et al. (2018), without training. In Mendonça et al. (2013), immediately after the training, the mean azimuth unsigned errors also decreased and reached a magnitude of 15.3° which is also comparable to the current results.

In the current study, without any familiarization, and with the three visual-to-auditory encodings, participants were able to discriminate the different azimuths as suggested by gains higher than the optimal value of 1.0. After the familiarization, and with the three visual-to-auditory encodings, participants were able to localize the azimuth of the target with average azimuth gains comprised between 1.23 and 1.41 which were higher than the null value and than the optimal gain 1.0. It shows that the sound spatialization method used in the current study based on HRTFs from the CIPIC database (Algazi et al., 2001a) partly reproduced the natural cues used in free-field sound azimuth localization. These results are not surprising since azimuth is mainly conveyed through binaural cues including the Interaural Level Difference (ILD) and the Interaural Time Difference (ITD) that reflect audio signal differences between the two ears. ITD is mainly used when the spectral content of the audio signal does not include frequencies higher than 1,500 Hz and ILD is mainly used for frequencies higher than 3,000 Hz (Blauert, 1996). The used frequencies ranged from 250 Hz to about 1,500 Hz with the Monotonic encoding, which is in a frequency domain where ITDs are mainly used to perceive the azimuth. With the Harmonic encoding, that added 2 octaves, the frequency range was between 250 and 6,000 Hz which already contains the ILD frequency domain. The Noise encoding with the broadband sound allows both cues (ITD and ILD) to be fully used, which can theoretically improve azimuth localization accuracy in comparison with sounds with a lower spectral complexity, as previously shown in Morikawa and Hirahara (2013). However, in the current study, these drastic changes in the spectrum did not strongly affect the participants' abilities, and the response patterns were similar. In other words, whatever the spectral complexity of the sound used in the encoding (white noise, complex tones or pure tones), binaural cues could be perceived and interpreted by the participants. It can be noticed that azimuth accuracy seems slightly higher with the two pitch-based encodings (the Harmonic and Monotonic encodings) in comparison with the spatialization-based only encoding (the Noise encoding). We did not find similar results in the scientific literature. This facilitation effect could result from a decrease in the cognitive load when the elevation is conveyed through the pitch modulation. As mentioned above, the pitch-based encodings seem more intuitive to localize the elevation, therefore it should globally decrease the cognitive load and thus facilitate the processing of the remaining dimension (i.e., the azimuth dimension). This effect does not seem to drastically shape the results and remains to be confirmed by other experiments.

The participants tended to overestimate the lateral position of the lateral targets with the three visual-to-auditory encodings: a shift to the left for targets on the left, and a shift to the right for targets on the right. Some studies also showed an overestimation pattern of lateral sound sources while using non-individualized HRTFs (Wenzel et al., 1993), in a virtual environment while being blindfolded (Ahrens et al., 2019), using ambisonics (Huisman et al., 2021), and even with real sound sources (Oldfield and Parker, 1984; Makous and Middlebrooks, 1990). A possibility to decrease this overestimation might be to rescale the used HRTF positions to fit to the perceived ones. For example one could rescale the azimuth angles of the HRTFs database to compensate for the non-linear shape that was measured as the perceived ones and measure if it could linearize the response profile.

**Figure 7:**
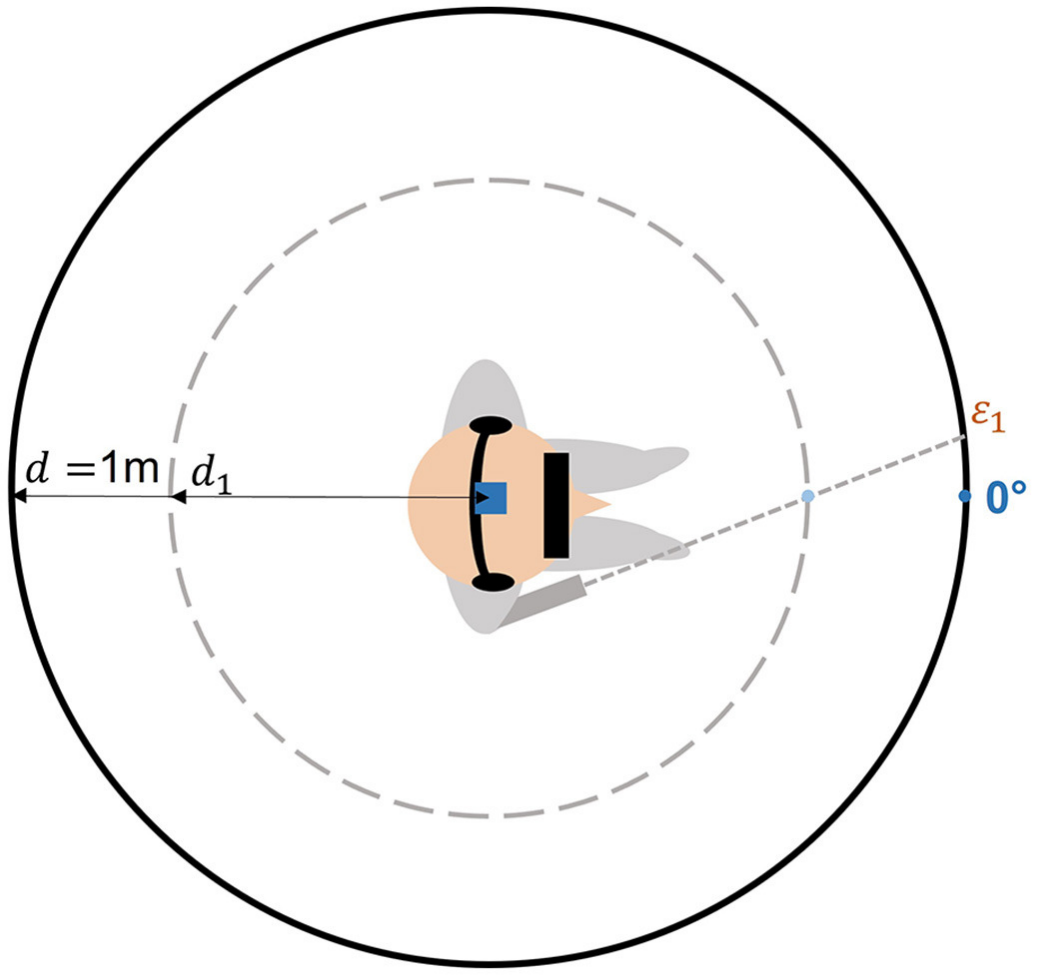
Left shift scheme. If the participant perceived the distance of the target closer than the real target distance (*d*_1_ instead of *d* = 1m), it might induce an increase of the leftward bias (ε_1_).

The participants also tended to localize the targets with a leftward bias between −1.2 and −5.03° in average. This systematic error might be due to a wrong auditory localization but also to a misperception of target distance. Geometrical considerations shows that an underestimation of the distance of the sound source would generate a leftward bias as we see in the current results. Since no indication concerning the sound distance was given, the participants could estimate that the sound sources were located closer than one meter. Figure 7 shows the effect of a misperception of target distance on the azimuth localization. However, for the same reason, a distance underestimation would have cause an overestimation of the elevation perception, which we did not measure.

### 4.3. A fast improvement in object localization performance

#### 4.3.1. A short but active familiarization method

After a first practice followed by a very short familiarization, participants' abilities to localize an object with the visual-to-auditory SSD were improved. The elevation gains were improved for all the encodings (especially for pitch-based ones), and for the azimuth, the decrease in the lateral overshoot suggests that the interpretation of acoustic cues provided by the ILD and ITD for the azimuth was improved. Since no feedback was given during the first practice, it can be supposed that the familiarization session mainly contributed to acquire sensorimotor contingencies (Auvray, 2004) through the mean of an audio-motor calibration (Aytekin et al., 2008).

In order to avoid a too long experimental session, we used a short audio-motor familiarization session (60 s) during which participants were active by controlling the position of the target, which is known to improve the positive effect of the training (Aytekin et al., 2008; Hüg et al., 2022). Other familiarization methods have been studied and have shown improvements in the use of SSDs. For example, prior to the experimental task, some studies simultaneously displayed to participants an image and its equivalent soundscape (Ambard et al., 2015; Buchs et al., 2021). In another study (Auvray et al., 2007), participants were enrolled in an intensive training of 3 h. Using only a verbal explanation of the visual-to-auditory encoding scheme as been shown to be efficient to understand the main principles of the encoding scheme (Kim and Zatorre, 2008; Buchs et al., 2021; Scalvini et al., 2022). The aim of the current study was not to directly investigate the effect of a short and active familiarization method on localization performance but it shows that a short practice might be sufficient to acquire the sensorimotor contingencies. The effect of the familiarization remains to be clearly assessed by comparing the efficiency of the existing methods with control conditions in order to optimize the SSD learning.

#### 4.3.2. Calibration of the auditory space improves localization abilities

In the current study, participants were not aware of the size of the VAS neither that the head tracker was associated with a virtual camera capturing and converting into sounds a limited portion of the virtual scene in front of them. They only knew that the virtual target would appear at random locations in their front-field at different azimuth and elevation locations. As a consequence, they also did not know the spatial boundaries of the space where the target could be heard. After a short practice, the participants were able to build an accurate mental spatial representation of the virtual space where the visual-to-auditory encoding took place. For instance, the downward bias in elevation decreased after the familiarization session, suggesting that participants learned that the VAS was at a higher location. Also the decrease of the overestimation pattern in azimuth suggests that participants learned that the lateral VAS boundaries were closer.

It has to be noticed that the size of the VAS has an influence on the localization accuracy. The biggest the VAS is, the higher the localization error might be. Restricting the field of view of the camera would result in a smaller possible space in which an heard target could be placed, thus resulting in a lower angular error, but as a counterpart, it would cover a smaller subpart of the front-field without moving the head. For instance, for a target placed in a central position, a random pointing in a VAS with a field of view of 45 × 45° (azimuth × elevation) would result in an error in azimuth and elevation with a standard deviation 2 times lower than with a field of view of 90 × 90° while covering a space 4 times smaller. Studying the effect of various VAS sizes in a target localization task in which the user can freely move the head to point to a target as fast as possible would probably give some insights about the optimal VAS size. However, in ecological contexts, a large VAS size would have the advantage of providing auditory information about obstacles placed with a larger eccentricity with respect to the forward direction of the head. For this reason, in a real context of use, this parameter should probably be customizable according to the habit of use.

## 5. Conclusion

Long trainings are required to master a visual-to-auditory SSD (Kristjánsson et al., 2016) because the used visual-to-auditory encodings are not enough intuitive (Hamilton-Fletcher et al., 2016b). In our study, we investigated several visual-to-auditory encodings in order to develop a SSD whose auditory information could quickly be interpreted to localize obstacles. In line with previous studies, our results suggest that a visual-to-auditory SSD based on the creation of a VAS is efficient to convey visuo-spatial information about azimuth through soundscapes. Our study shows that a pitch-based elevation mapping can be easily learn to compensate for elevation localization impairments due to the use of non-individualized HRTFs in the creation process of the VAS. Despite a very short period of practice, the participants were able to improve their interpretation of the used acoustic cues both for the azimuth and the elevation encoding schemes.

## Data availability statement

The raw data supporting the conclusions of this article will be made available by the authors, without undue reservation. The original contributions presented in the study will be made available in the following link: http://leadserv.u-bourgogne.fr/en/members/maxime-ambard/pages/cross-modal-correspondance-enhances-elevation-localization. Further questions should be directed to the corresponding author.

## Ethics statement

The studies involving human participants were reviewed and approved by Comité d'Ethique pour la Recherche de Université Bourgogne Franche-Comté. The patients/participants provided their written informed consent to participate in this study.

## Author contributions

CB and MA contributed to conception and design of the study and interpreted the data. CB executed the study and was responsible for data analysis and wrote the first draft of the manuscript in closed collaboration with MA. FS, CM, and JD provided important feedback. All authors have read, approved the manuscript, and contributed substantially to it.

## Supplementary material

Supplementary Figure 1 Elevation response position as a function of target elevation for each participant of the Monotonic group. Mean elevation response positions (in degree) before **(left)** and after **(right)** are represented separately for the Noise (blue squares) and the Monotonic (orange circles) encodings. Error bars shows standard error of elevation response position. Solid lines represent the elevation gains with the Noise (blue) and Monotonic (orange) encodings. Black dashed lines indicate the optimal elevation gain 1.0.

Supplementary Figure 2 Elevation response position as a function of target elevation for each participant of the Harmonic group. Mean elevation response positions (in degree) before **(left)** and after **(right)** are represented separately for the Noise (blue squares) and the Harmonic (red circles) encodings. Error bars shows standard error of elevation response position. Solid lines represent the elevation gains with the Noise (blue) and Harmonic (red) encodings. Black dashed lines indicate the optimal elevation gain 1.0.

Supplementary Figure 3 Azimuth response position as a function of target azimuth for each participant of the Monotonic group. Mean azimuth response positions (in degree) before **(left)** and after **(right)** are represented separately for the Noise (blue squares) and the Monotonic (orange circles) encodings. Error bars shows standard error of azimuth response position. Solid lines represent the azimuth gains with the Noise (blue) and Monotonic (orange) encodings. Black dashed lines indicate the optimal azimuth gain 1.0.

Supplementary Figure 4 Azimuth response position as a function of target azimuth for each participant of the Harmonic group. Mean azimuth response positions (in degree) before **(left)** and after **(right)** are represented separately for the Noise (blue squares) and the Harmonic (red circles) encodings. Error bars shows standard error of azimuth response position. Solid lines represent the azimuth gains with the Noise (blue) and Harmonic (red) encodings. Black dashed lines indicate the optimal azimuth gain 1.0.
