# Supplementary figures and images for: Cross-modal correspondence enhances elevation localization in visual-to-auditory sensory substitution
Source: Front Psychol. 2023 Jan 26;14:1079998. doi: 10.3389/fpsyg.2023.1079998 (PMC9909421; doi:10.3389/fpsyg.2023.1079998)

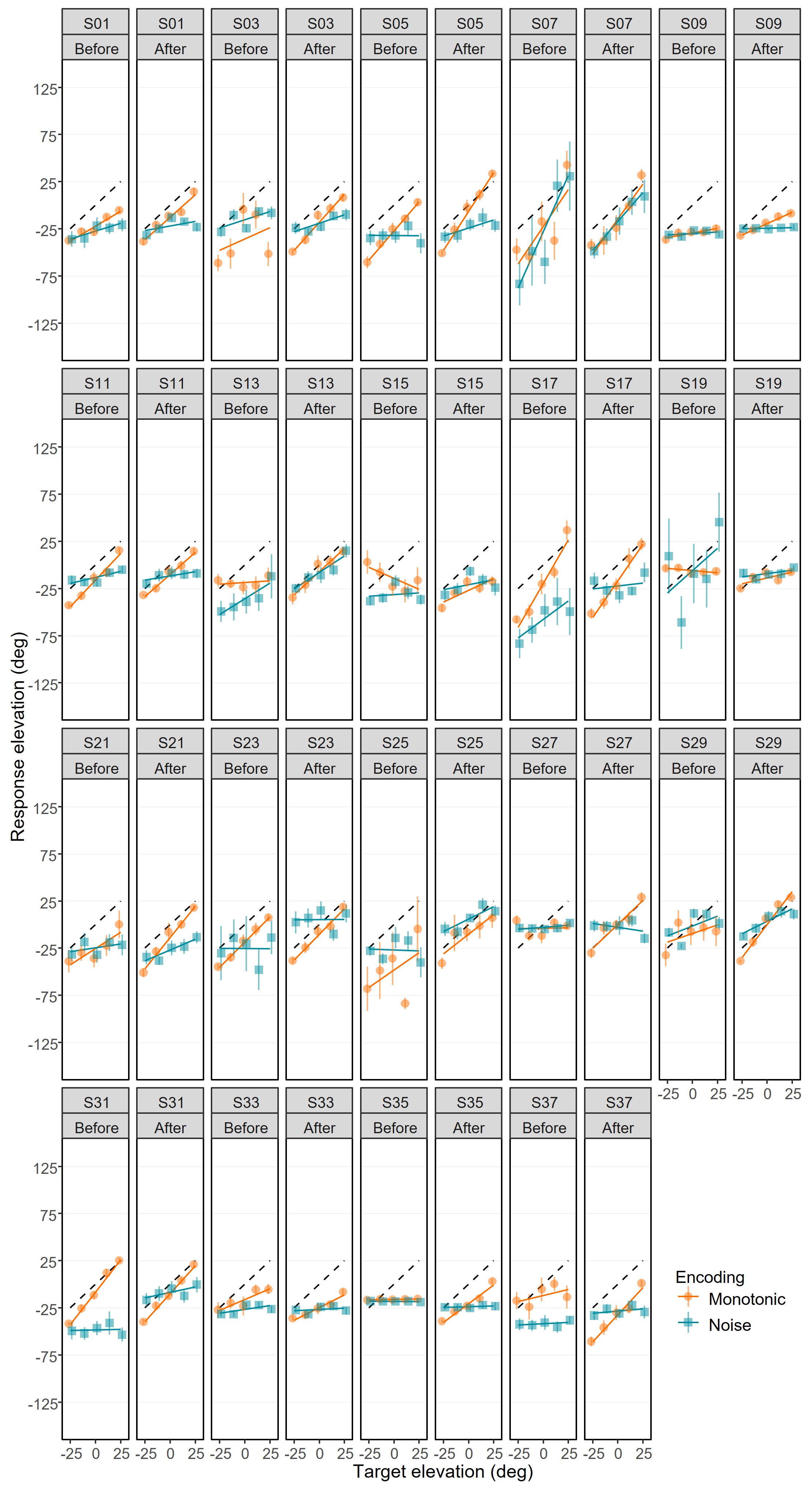

Supplement: Supplementary Figure 1 — Elevation response position as a function of target elevation for each participant of the Monotonic group. Mean elevation response positions (in degree) before (left) and after (right) are represented separately for the Noise (blue squares) and the Monotonic (orange circles) encodings. Error bars shows standard error of elevation response position. Solid lines represent the elevation gains with the Noise (blue) and Monotonic (orange) encodings. Black dashed lines indicate the optimal elevation gain 1.0. [file Image_1.jpg]

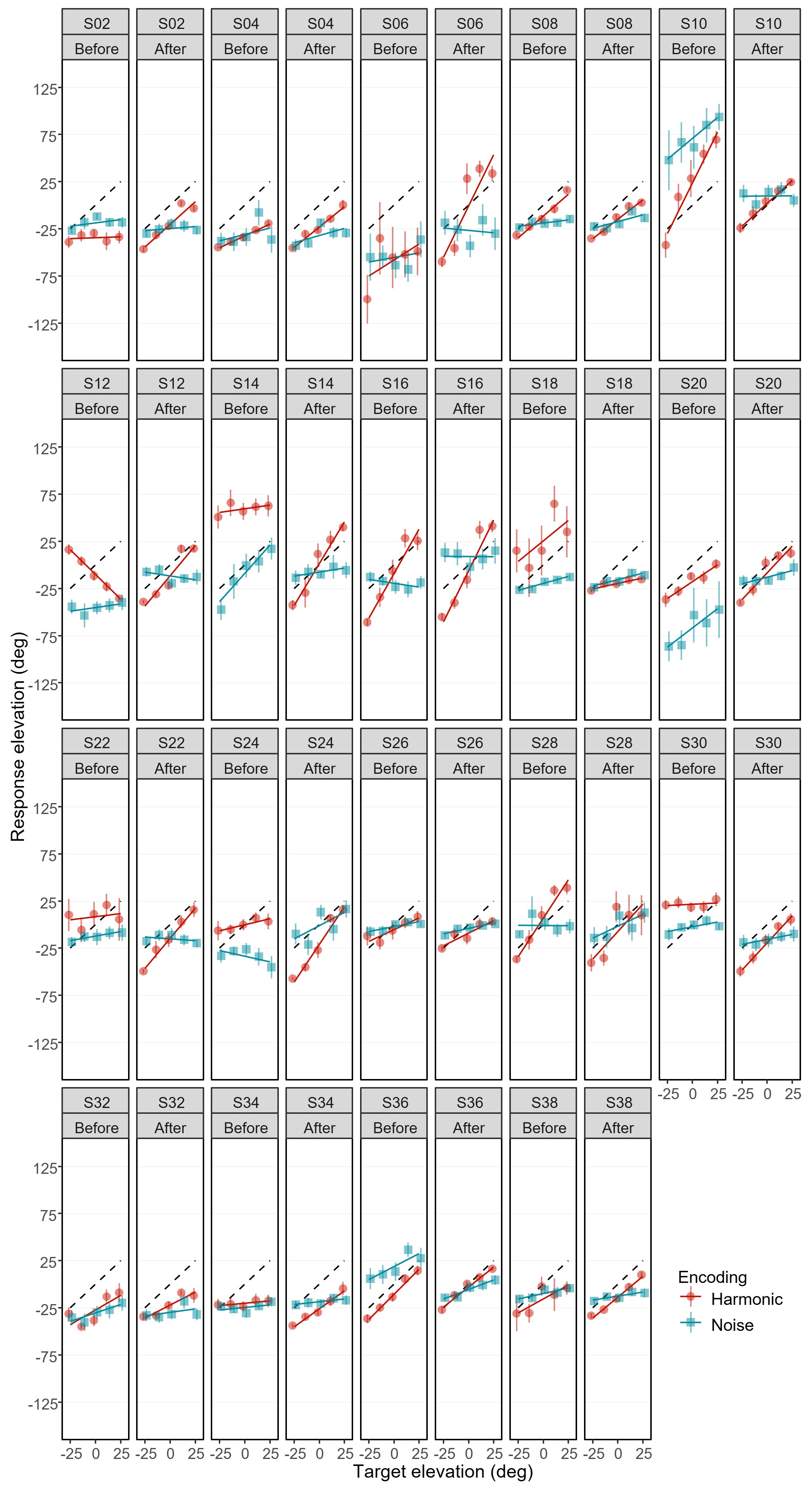

Supplement: Supplementary Figure 2 — Elevation response position as a function of target elevation for each participant of the Harmonic group. Mean elevation response positions (in degree) before (left) and after (right) are represented separately for the Noise (blue squares) and the Harmonic (red circles) encodings. Error bars shows standard error of elevation response position. Solid lines represent the elevation gains with the Noise (blue) and Harmonic (red) encodings. Black dashed lines indicate the optimal elevation gain 1.0. [file Image_2.jpg]

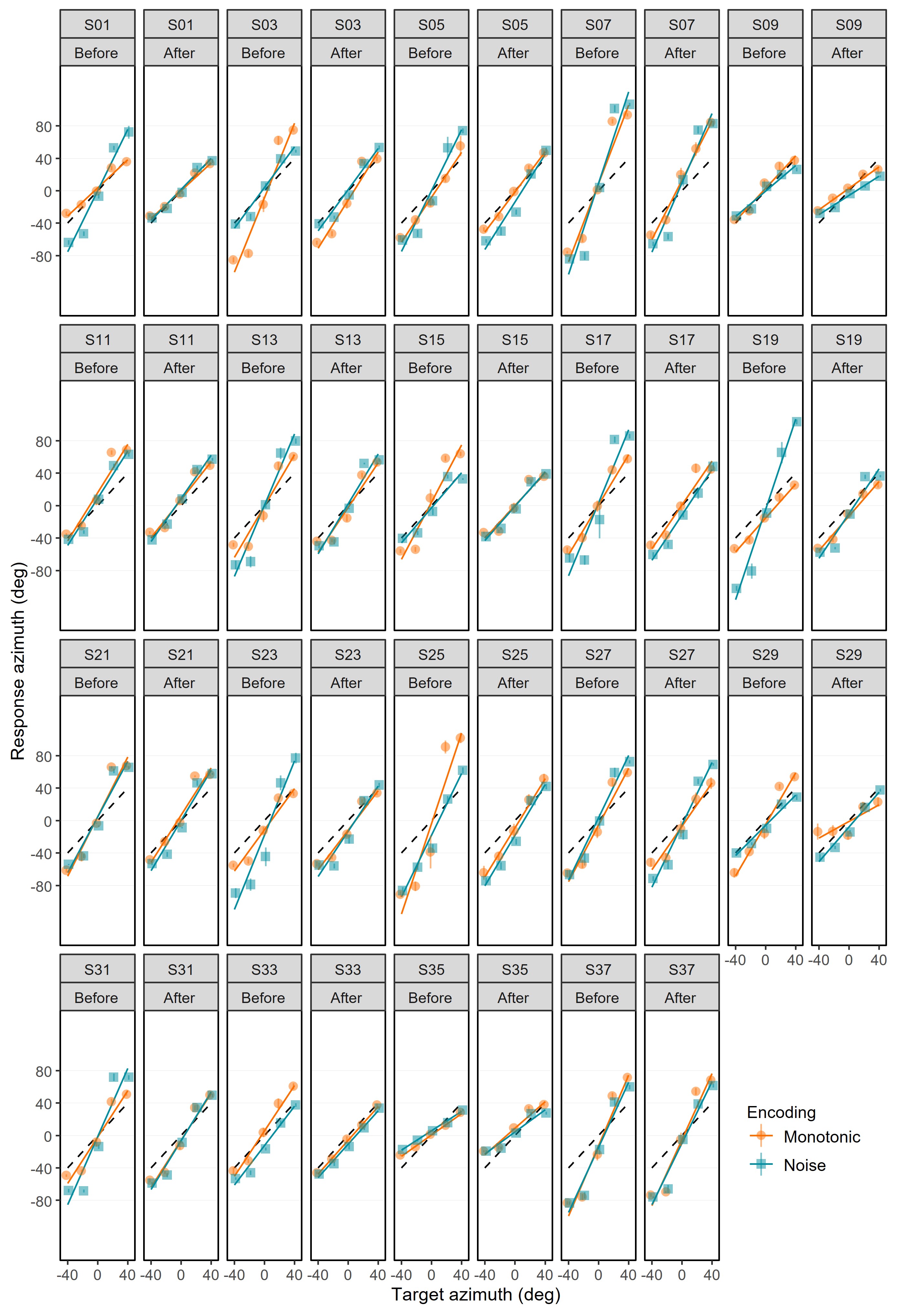

Supplement: Supplementary Figure 3 — Azimuth response position as a function of target azimuth for each participant of the Monotonic group. Mean azimuth response positions (in degree) before (left) and after (right) are represented separately for the Noise (blue squares) and the Monotonic (orange circles) encodings. Error bars shows standard error of azimuth response position. Solid lines represent the azimuth gains with the Noise (blue) and Monotonic (orange) encodings. Black dashed lines indicate the optimal azimuth gain 1.0. [file Image_3.jpg]

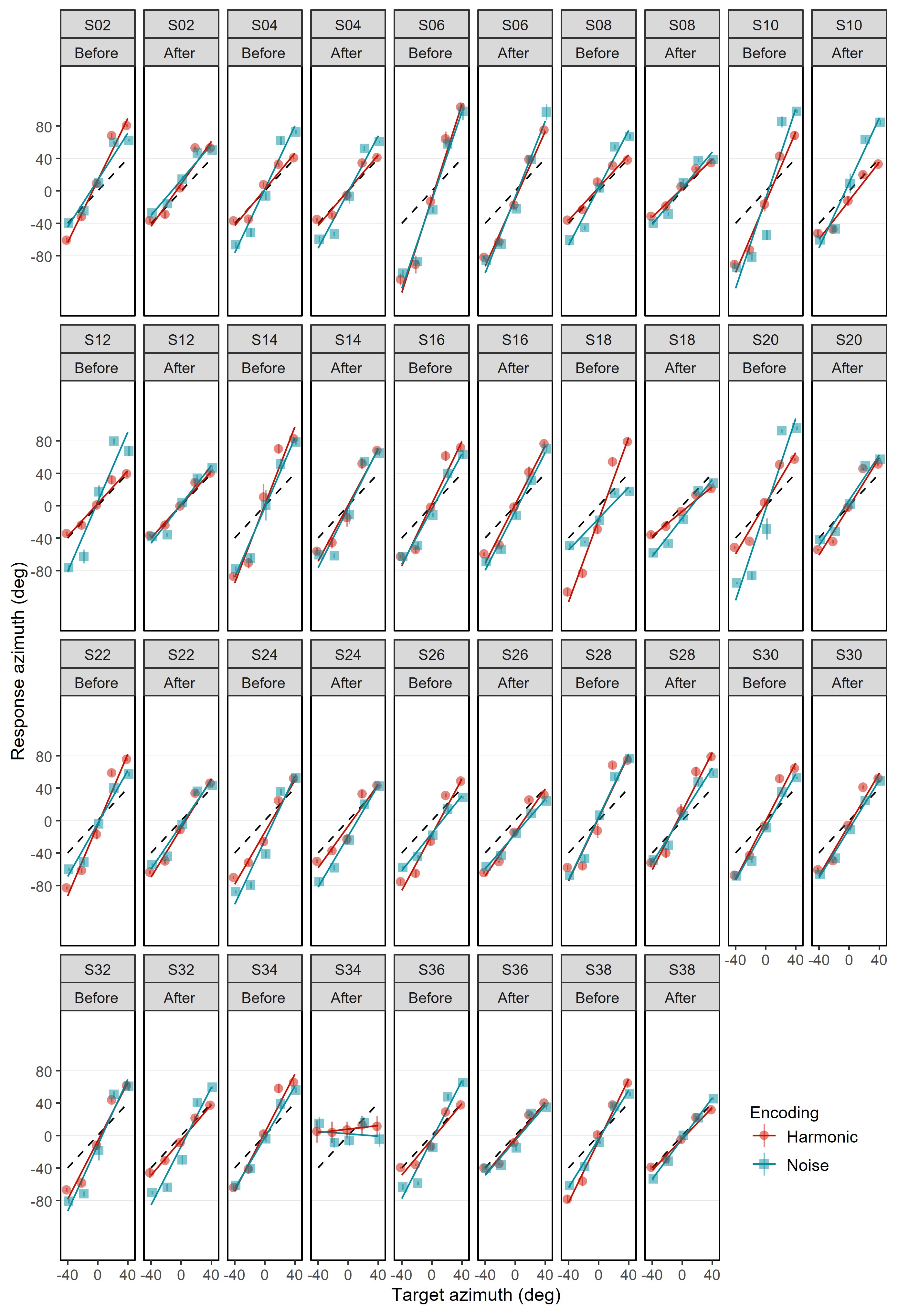

Supplement: Supplementary Figure 4 — Azimuth response position as a function of target azimuth for each participant of the Harmonic group. Mean azimuth response positions (in degree) before (left) and after (right) are represented separately for the Noise (blue squares) and the Harmonic (red circles) encodings. Error bars shows standard error of azimuth response position. Solid lines represent the azimuth gains with the Noise (blue) and Harmonic (red) encodings. Black dashed lines indicate the optimal azimuth gain 1.0. [file Image_4.jpg]
